# Dual Deletion of *Keap1* and *Rbpjκ* Genes in Liver Leads to Hepatomegaly and Hypercholesterolemia

**DOI:** 10.3390/ijms25094712

**Published:** 2024-04-26

**Authors:** Nobunao Wakabayashi, Yoko Yagishita, Tanvi Joshi, Thomas W. Kensler

**Affiliations:** 1 Translational Research Program, Fred Hutchinson Cancer Center, Seattle, WA 98109, USA; nwakabay@fredhutch.org (N.W.); yyagishi@fredhutch.orgtjoshi@fredhutch.org (T.J.); 2 Division of Endocrinology, Columbia University, New York, NY 10032, USA

**Keywords:** liver, cholesterol, cholestasis, hypercholesterolemia, KEAP1, NRF2, RBPJκ, NOTCH signaling

## Abstract

The hepatic deletion of Rbpjκ (*Rbpj^F/F^::AlbCre*) in the mouse leads to exhibition of the Alagille syndrome phenotype during early postnatal liver development with hyperlipidemia and cholestasis due to attenuated disruption of NOTCH signaling. Given the roles of NRF2 signaling in the regulation of lipid metabolism and bile ductal formation, it was anticipated that these symptoms could be alleviated by enhancing NRF2 signaling in the *Rbpj^F/F^::AlbCre* mouse by hepatic deletion of *Keap1* in compound *Keap1^F/F^::Rbpj^F/F^::AlbCre* mice. Unexpectedly, these mice developed higher hepatic and plasma cholesterol levels with more severe cholestatic liver damage during the pre-weaning period than in the *Rbpj^F/F^::AlbCre* mice. In addition, hypercholesterolemia and hepatic damage were sustained throughout the growth period unlike in the *Rbpj^F/F^::AlbCre* mouse. These enhanced abnormalities in lipid metabolism appear to be due to NRF2-dependent changes in gene expression related to cholesterol synthetic and subsequent bile acid production pathways. Notably, the hepatic expression of *Cyp1A7* and *Abcb11* genes involved in bile acid homeostasis was significantly reduced in *Keap1^F/F^::Rbpj^F/F^::AlbCre* compared to *Rbpj^F/F^::AlbCre* mice. The accumulation of liver cholesterol and the weakened capacity for bile excretion during the 3 pre-weaning weeks in the *Keap1^F/F^::Rbpj^F/F^::AlbCre* mice may aggravate hepatocellular damage level caused by both excessive cholesterol and residual bile acid toxicity in hepatocytes. These results indicate that a tuned balance of NOTCH and NRF2 signaling is of biological importance for early liver development after birth.

## 1. Introduction

Dyslipidemia and its related disorders, including metabolic-associated steatotic liver disease, are of expanding public health concern as levels of MASH incidence increase [1]. The liver plays a central role in these pathogenetic processes as the primary organ controlling cholesterol and subsequent bile acid and fatty acid metabolism. Impaired bile acid metabolism leading to cholestatic liver disease is often accompanied by serious hypercholesterolemia, affecting childhood development and nutrition. Alagille syndrome (AGS) is a rare hereditary cholestatic disorder initially caused by mutations in JAGGED1, a ligand gene product of the NOTCH signaling pathway, or its receptor gene, *NOTCH2* [2]. With AGS, cholestasis is thought to result from a paucity of intrahepatic bile ducts [3]. The disorder also gives rise to various extrahepatic pathological anomalies such as peripheral pulmonary stenosis, butterfly-like vertebrae, facial and ocular malformations and growth disturbances.

Recombination signal binding protein for immunoglobulin kappa j region (RBPJ) [4] is the practical effector for the transduction of NOTCH signaling. RBPJ provides direct DNA-binding capacity [5] when heterodimerized with the NOTCH intracellular domain, which becomes the transcription co-factor processed by γ-secretase following NOTCH–ligand association with the extracellular domain of NOTCH. RBPJ is essential for the activation/repression of NOTCH signaling [6]. Two lines of hepatic *Rbpj* deletion mice have been developed, which utilize different Cre-deletion strategies to regulate its expression by the albumin (*Alb*) promoter [7,8] and under the control of both the *Alb* promoter and alpha-fetoprotein enhancers (*Alfp*) [9]. Due to differences in the timing of Cre activation, while both lines of mice survive, the *Rbpj^F/F^::AlfpCre* mouse exhibits more severe cholestasis accompanied by growth retardation, whereas the *Rbpj^F/F^::AlbCre* mouse only manifests cholestasis during its pre-weaning period [10]. There are close resemblances in the phenotypes observed in hepatic *Notch2* or *Jagged1* deletion mice to those of *Rbpj*-Cre mice [10,11,12]. However, it is still under debate as to which mechanisms are involved in liver regeneration to restore liver cell mass and to maintain hepatic lipid homeostasis in the context of a cholestatic liver. Although the causative genes are established, the broad functions of NOTCH signaling, an evolutionarily conserved pathway regulating pleiotropic cellular events such as cell fate decisions including differentiation, proliferation, and regeneration, and hepatic lipid metabolism in this disease are still relatively unknown [13,14,15].

Nuclear factor erythroid 2-related factor 2 (NRF2) is a transcription factor belonging to the Cap’n’Collar subfamily bearing a basic leucine zipper structure that is expressed ubiquitously in tissues. The target genes of NRF2 were classified initially as xenobiotic detoxication enzymes and oxidant/redox scavenger enzymes such as NAD(P)H quinone dehydrogenase 1 (NQO1) [16]; however, now, a wide range of target genes affecting homeostasis and cell fate have been defined that include anti-inflammatory, metabolism, cancer onset/progression and cell-death with efferocytosis signaling and post-transcriptional regulators (miRNA) [17]. There are cis-elements referred to as antioxidant responsive elements (AREs) on the gene regulatory region of the target genes. NRF2 directly binds to the ARE as dimers with small musculoaponeurotic fibrosarcoma proteins (MafF, MafG, and MafK) [16,18]. The resultant assembly provides the transcription machinery to output positive or negative responses that can be cell-type-dependent [19].

Post-translational regulation of NRF2 by ubiquitin-proteasomal degradation is assisted by multiple molecular chaperones. This regulation is very important for the maintenance of cell homeostasis. Kelch-like ECH associated protein 1 (KEAP1) [20] is the highest-affinity chaperon to NRF2 and is localized principally in the cytosol in conjunction with CULLIN 3 and other accessory proteins, forming an NRF2 degron that mediates the effective degradation of NRF2 [21]. The biological significance of this molecular interaction has been confirmed following the establishment of *Nrf2*, *Keap1*, and *Nrf2-Keap1* dual-disrupted mice. These models include *Keap1* constitutive knockout mice, which exhibit postnatal lethality before weaning by malnutrition due to NRF2-driven esophageal constriction, heterozygotes that survive, as well as *Keap1* knockdown mice (*Keap1^F/F^*) [22] with constitutive hypomorphic *Keap1* expression in the absence of Cre expression. Notably, the phenotype evoked by *Keap1* deletion was reversed by the disruption of *Nrf2* in *Nrf2-Keap1* dual-deletion mice [23].

It has been reported that NRF2 contributes to the expression of genes involved in lipid metabolism, including the cholesterol metabolic system [24,25,26,27]. We observed previously that *Keap1^F/F^::AlbCre* mice exhibited greater microbranching of the intrahepatic bile ducts in the liver lobes of mice than in control as well as in *Rosa^NICD/+^::AlbCre* mice, which express higher NOTCH signaling in the liver [28]. Therefore, we considered whether cholestasis mediated by the depletion of NOTCH signaling in the *Rbpj^F/F^::AlbCre* mouse liver could be rescued by activating lipid metabolic pathways through enhanced NRF2 signaling. For this purpose, *Keap1^F/F^::Rbpj^F/F^::AlbCre*, *Nrf2^F/F^::Keap1^F/F^::Rbpj^F/F^::AlbCre* or *Nrf2*^−/−^:: *Keap1^F/F^::Rbpj^F/F^::AlbCre* mouse lines were established and compared with the *Rbpj^F/F^::AlbCre* mouse with a focus on lipid metabolism. Against the expectations, the *Keap1^F/F^::Rbpj^F/F^::AlbCre* mouse developed higher hepatic and plasma cholesterol levels, and exacerbated hepatic damage accompanied with hepatomegaly during the pre-weaning 3 W period, than observed in the *Rbpj^F/F^::AlbCre* mouse. Moreover, hypercholesterolemia and hepatic damage were sustained throughout the growth period (to at least 9 W) unlike in the *Rbpj^F/F^::AlbCre* mouse. Interestingly, this physiological phenomenon was reversed in the *Nrf2^F/F^::Keap1^F/F^::Rbpj^F/F^::AlbCre* mouse, particularly evident after weaning (4 W, 9 W). By comparisons of the hepatic expression of lipids and subsequent bile acid synthesis-related genes among *Rbpj^F/F^::AlbCre*, *Keap1^F/F^::Rbpj^F/F^::AlbCre* and *Nrf2^F/F^::Keap1^F/F^::Rbpj^F/F^::AlbCre* mice, it was observed that genes important in bile acid production and elimination, *Cyp7A1* and *Abcb11*, were reduced significantly in the *Keap1^F/F^::Rbpj^F/F^::AlbCre* mice at 3 W of age. The higher accumulation of liver cholesterol and weakening of bile excretion during the pre-weaning period in *Keap1^F/F^::Rbpj^F/F^::AlbCre* mice likely enhanced hepatocellular damage through both excessive cholesterol and residual bile toxicity in hepatocytes compared to the *Rbpj^F/F^::AlbCre* mice.

## 2. Results

### 2.1. Keap1 Deletion in Rbpj^F/F^::AlbCre Mice Elicits More Severe, Persistent Hepatic Toxicity and Hepatomegaly than Observed in Rbpj^F/F^::AlbCre Mice

**Figure 1:**
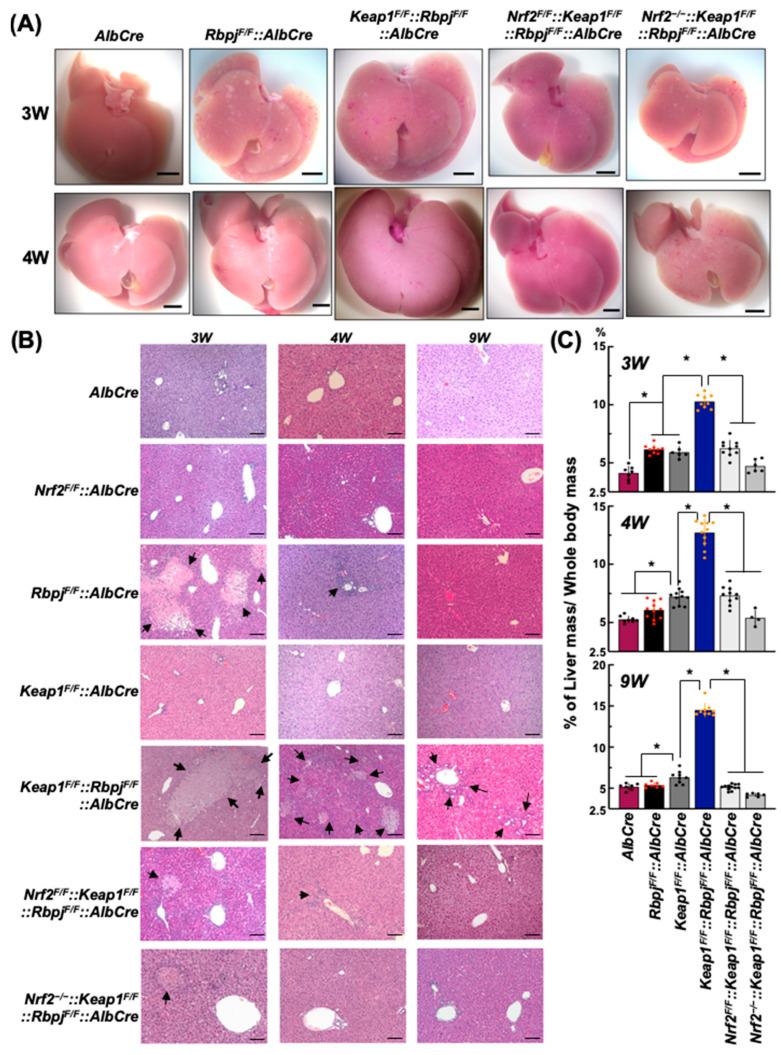
Changes in liver morphology among the genotypes. (**A**) Representative livers from 3 W- and 4 W-old males. The scale bar = 5 mm. (**B**) H&E-stained sections from the central lobe of each genotype mouse at 3 W, 4 W and 9 W of age. Arrows indicate the degeneration and damage produced by cholestasis. The scale bar = 100 μm. (**C**) Size of the livers presented as % of whole-body mass. N = 4–12. * *p* < 0.05, by Tukey’s (highlighted by red or orange datapoints).

Hepatoblast lineage-specific *Rbpjκ* (*Rbpj*) deletion using transgene *AlbCre* expression in mice causes a diminution of intrahepatic bile duct development in the early postnatal period [29,30]. This disruption leads to severe cholestasis followed by hepatic necrosis and fibrosis within infancy, as seen in humans with AGS [3,10], which is an autosomal dominant disorder evoked by point mutations in *JAG1* and less commonly *NOTCH2* genes [2]. The initial phenotype appears in *Rbpj^F/F^::AlbCre* mice, as seen in Figure 1A,B. Hepato-necrotic areas are observed as white spots with presumably hepatocyte bursting traces and efferocytic areas infiltrated with inflammatory cells (Figure 1A, Supplemental Figure S1) and confirmed by observing H&E-stained liver sections (Figure 1B, Supplemental Figure S2). The majority of necrotic areas are observed in zone 2 of hepatic *Rbpj*-disrupted mice and their related compound-disrupted mice (*Rbpj^F/F^::AlbCre*, *Keap1^F/F^::Rbpj^F/F^::AlbCre*, *Nrf2^F/F^::Keap1^F/F^::Rbpj^F/F^::AlbCre or Nrf2*^−/−^*::Keap1^F/F^::Rbpj^F/F^::AlbCre*) but never in the control mice used in establishing these genotypes (*Wild-type*, *AlbCre*, *Keap1^F/F^::AlbCre*, *Nrf2^F/F^::AlbCre or Nrf2*^−/−^*::AlbCre*, Supplemental Figure S3). Interestingly, the severe necrotic areas seen in 3 W-old mice were observed as very small or low-frequency traces in *Rbpj^F/F^::AlbCre* mice by 4 W postnatally. These lesions are not seen at 9 W of age. Only in 9 W-old *Keap1^F/F^::Rbpj^F/F^::AlbCre* mice are the traces of putative necrotic areas still observed. Thus, cholestasis in the *Rbpj^F/F^::AlbCre* mice is transient through the course of the pre-weaning period, but persists throughout the growth stage of *Keap1^F/F^::Rbpj^F/F^::AlbCre* mice.

From weaning (3 W) to 4 W and 9 W of age, the percent of whole liver mass per whole body mass of each mouse was measured (Figure 1C). Except for *Nrf2*^−/−^*::Keap1^F/F^::Rbpj^F/F^::AlbCre* livers, mice of all genotypes that included hepatic *Rbpjk* disruption exhibited enlarged livers compared to control *AlbCre* mice at 3 W. However, by 9 W, only in *Keap1^F/F^::Rbpj^F/F^::AlbCre* mice were livers larger than age-matched control *AlbCre* mice amongst the *Rbpjk* disruption genotypes. As in previous reports [28], *Keap1^F/F^::AlbCre* mice reproducibly exhibited enlarged livers. The sizes of livers of *Keap1^F/F^::Rbpj^F/F^::AlbCre* mice were approximately double that of *Rbpj^F/F^::AlbCre* and *Keap1^F/F^::AlbCre* mice at 3 W of age. This proportional size difference remained at all time points of observation. Notably, disruption of *Nrf2* in the *Nrf2^F/F^::Keap1^F/F^::Rbpj^F/F^::AlbCre* mice abrogated the amplifying effect of *Keap1* disruption on the *Rbpjκ* phenotype at all time points. The proportional liver sizes of the *AlbCre*, *Rbpj^F/F^::AlbCre* and *Nrf2*^−/−^*::Keap1^F/F^::Rbpj^F/F^::AlbCre* mice were similar (Figure 1C). Therefore, it is likely that the enhanced hepatomegaly seen in *Keap1^F/F^::Rbpj^F/F^::AlbCre* mice arose from excess NRF2 signaling following cholestasis induced by the loss of the *Rbpjκ* gene.

### 2.2. Plasma Clinical Chemistry and Hepatic Lipid Analyses in Keap1^F/F^::Rbpj^F/F^::AlbCre Mice and Modified Genotypes during the Growth Period

**Figure 2:**
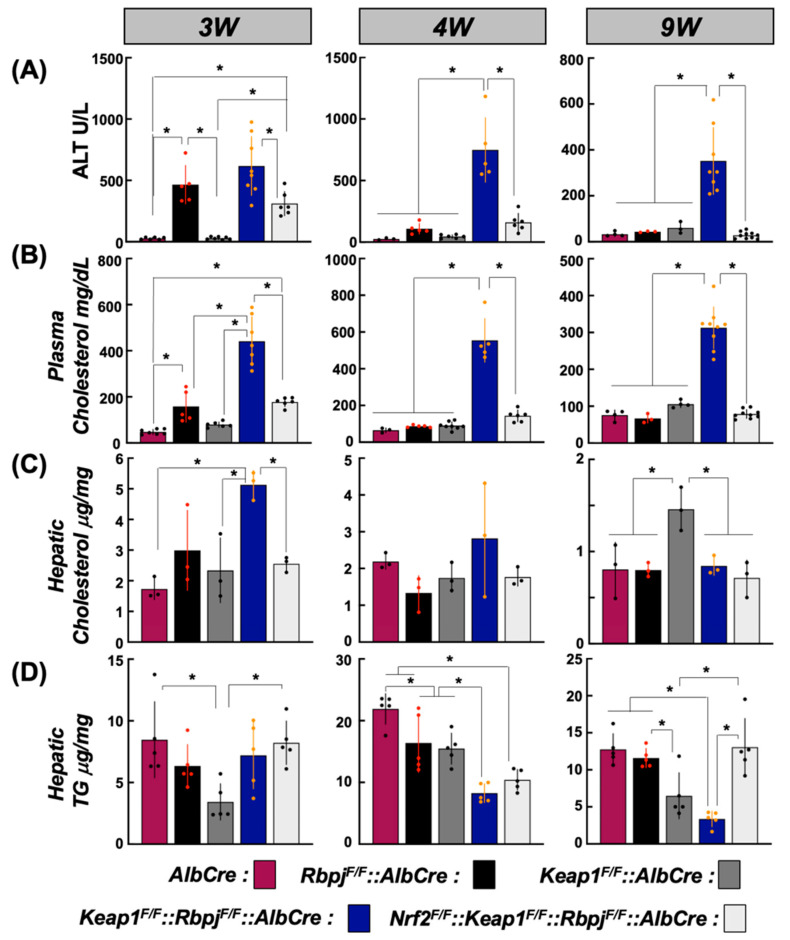
Plasma and hepatic biochemical analyses in each genotype mouse at 3 W, 4 W and 9 W of age. Plasma ALT and cholesterol levels are shown in (**A**) N = 3–10, (**B**) N = 3–9 and hepatic cholesterol and triglyceride levels are shown in (**C**) N = 3, (**D**) N = 5, respectively. * *p* < 0.05, by Tukey’s (highlighted by red or orange datapoints).

To quantify the hepatic damage induced by cholestasis in hepatic *Rbpjκ*-disrupted and related mice by physiological measures, assays of plasma clinical chemistry parameters were conducted. As anticipated from the histochemical analyses, plasma alanine transaminase (ALT) activity from *Rbpj^F/F^::AlbCre* mice was remarkable higher than control genotype mice (*AlbCre* and *Keap1^F/F^::AlbCre* mice that did not suffer cholestasis) and elevated to a similar degree in *Keap1^F/F^::Rbpj^F/F^::AlbCre* mice at 3 W. ALT returned to the levels of control mice by 4 W and 9 W of age in *Rbpj^F/F^::AlbCre* mice. However, plasma from *Keap1^F/F^::Rbpj^F/F^::AlbCre* mice showed elevated ALT activity that persisted to 9 W (Figure 2A). This temporal transition pattern seen in ALT activity also was observed in plasma AST, ALP activities, total bilirubin (Supplemental Figure S4) and cholesterol levels (Figure 2B).

Interestingly, plasma from *Nrf2^F/F^::Keap1^F/F^::Rbpj^F/F^::AlbCre* mice exhibited plasma ALT activity and cholesterol levels with the temporal transition similar to that in *Rbpj^F/F^::AlbCre* mice. Thus, it is likely that *Keap1* disruption from the livers of *Rbpj^F/F^::AlbCre* mice transformed the transient cholestatic phenotype in the *Rbpj^F/F^::AlbCre* mice to the persistent one observed at 9 W. Therefore, hepatic cholesterol and triglyceride levels in each mouse liver, which are the major de novo lipids synthesized in the *AlbCre* transgene-expressing organ, were examined at the 3 W, 4 W and 9 W timepoints (Figure 2C,D). At all time points, the hepatic triglyceride levels were lower or similar to the level of triglycerides in the *AlbCre* control mice (Figure 2D). Hepatic cholesterol levels were reflected in the plasma cholesterol levels across the genotypes at 3 W of age. However, they were restored to almost equivalent levels at 4 W and 9 W with only *Keap1^F/F^::AlbCre* mice showing 1.5-fold higher hepatic cholesterol levels than the other genotypes at 9 W (Figure 2C). Therefore, the phenotypical hypercholesterolemia detected through the dual disruption of *Keap1* and *Rbpjκ* genes from hepatocytes is hypothesized to be due to effects through anomalous lipid metabolism during the time of lactation feeding of the postnatal pups.

### 2.3. Altered Expression of Hepatic De Novo Lipid Synthesis Genes at the Transcriptional Level in Early Development among Key Genotypes

**Figure 3:**
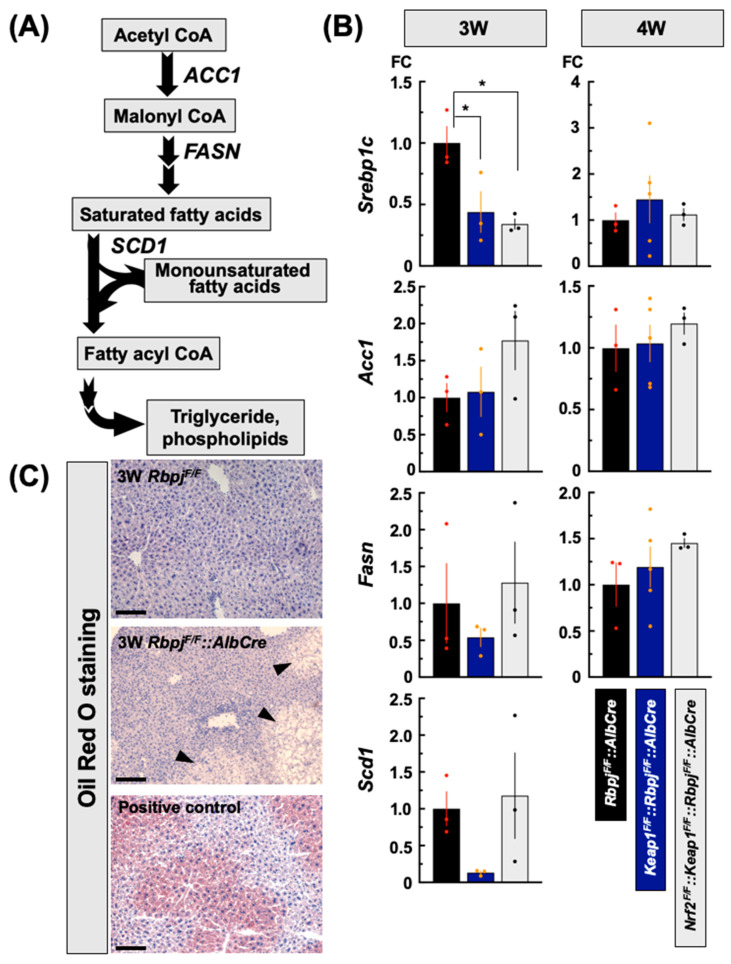
Fatty acid and triglyceride synthesis transcripts, which are mainly regulated by SREBP1C in liver, changed in each genotype. (**A**) SREBP1C-related fatty acid synthesis and its metabolic pathway. (**B**) Relative expression levels at 3 W and 4 W of age for *Srebp1c*, *Acc1*, *Fasn* and *Scd1*. The Y axis indicates the level of fold change (FC) where gene expression in *Rbpj^F/F^*::*AlbCre* mice was set as 1. N = 3–5 * *p* < 0.05 by Tukey’s (highlighted by red or orange datapoints). (**C**) The degenerated foci observed in *Rbpj^F/F^::AlbCre* liver were confirmed by oil red O staining. Arrow heads indicate degenerated regions in the liver. The scale bar = 100 μm.

Dyslipidemia is caused by a disruption of lipid metabolic homeostasis and dysfunction of the sterol regulatory element-binding proteins (SREBPs) that have a central role in this process [31]. In the liver, SREBP1c preferentially enhances the transcription of genes required for fatty acid synthesis (Figure 3A) but not cholesterol synthesis. By contrast, SREBP2 preferentially contributes to regulation of gene expression controlling cholesterol synthes is [32]. Therefore, the expression of genes influenced by the SREBP1c and SREBP2 systems, as well as the subsequent bile acid synthesis pathway, were examined in the livers of the mouse genotypes.

#### 2.3.1. Effect on De Novo Fatty Acid Synthesis-Related Gene Transcripts

To explore the key genes responsible for the severe phenotypes, gene expressions related to hepatic lipid metabolism before and after weaning were measured in the three genotypes that exhibited the different trajectories of cholestatic severity and persistence: *Rbpj^F/F^::AlbCre*, *Keap1^F/F^::Rbpj^F/F^::AlbCre* and *Nrf2^F/F^::Keap1^F/F^::Rbpj^F/F^::AlbCre*. *Rbpj^F/F^::AlbCre* mice did not accumulate hepatic triglycerides (Figure 2D), which was confirmed by oil red O staining of 3 W *Rbpj^F/F^::AlbCre* mice even in the partially swollen areas where cells were not disrupted by necrosis. Levels were equivalent to the level of *Rbpj^F/F^* mice in which the *Rbpjκ* gene was not disrupted (Figure 3C, Supplemental Figure S5).

*SREBP1c* transcripts in *Rbpj^F/F^::AlbCre* mice were ~1.5- to 2-fold higher than in *Keap1^F/F^::Rbpj^F/F^::AlbCre* and *Nrf2^F/F^::Keap1^F/F^::Rbpj^F/F^::AlbCre* mice. (Figure 3B) at 3 W; however, the transcripts reverted to similar levels at 4 W in these genotypes that developed cholestasis (Figure 3B). Expression of fatty acid synthesis-related genes such as *Acc1*, *Fasn* and *Scd1* incompletely followed the pattern of *SREBP1c* transcripts in *Rbpj^F/F^::AlbCre* mice at 3 W. *Fasn* and *Scd1* transcript levels were lower in *Keap1^F/F^::Rbpj^F/F^::AlbCre* mice but restored to *Rbpj^F/F^::AlbCre* levels in *Nrf2^F/F^::Keap1^F/F^::Rbpj^F/F^::AlbCre* mice. *Acc1* transcript levels did not change between *Rbpj^F/F^::AlbCre* and *Keap1^F/F^::Rbpj^F/F^::AlbCre* mice, but were elevated in the livers *of Nrf2^F/F^::Keap1^F/F^::Rbpj^F/F^::AlbCre* mice. Levels of these gene transcripts returned to equivalent levels at 4 W in these three genotypes (Figure 3B).

#### 2.3.2. Effect on De Novo Cholesterol Synthesis-Related Gene Transcripts

**Figure 4:**
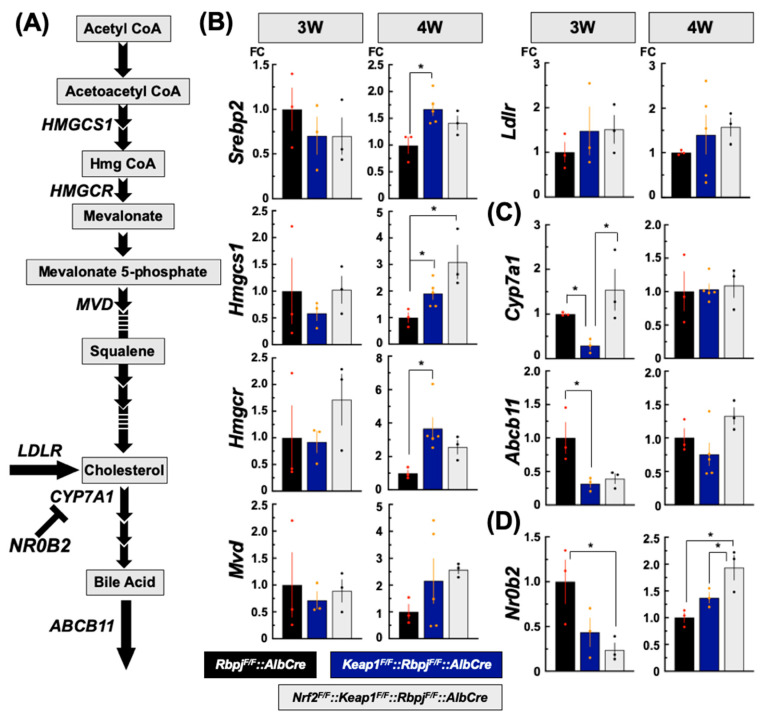
Cholesterol synthesis- and metabolism-related gene expression changes in each genotype. SREBP2-related metabolic and subsequent bile acid synthesis pathways are diagrammed in (**A**). De novo cholesterol synthesis, bile acid synthesis and efflux, and *NR0B2* relative gene expression levels at 3 W- and 4 W-old are shown in (**B**–**D**), respectively. The Y axis indicates the level of fold change (FC) where gene expression in *Rbpj^F/F^*::*AlbCre* mice was set as 1. N = 3–5, * *p*< 0.05 by Tukey’s (highlighted by red or orange datapoints).

Interestingly, in all liver genotypes, transcript levels of *Srebp2* and its target genes (Figure 4A) [32], *Hmgcs1*, *Hmgcr*, *Mvd* and *Ldlr*, were similar among the mice at 3 W (Figure 4B), even though plasma and hepatic cholesterol levels were much higher in the *Nrf2^F/F^::Keap1^F/F^::Rbpj^F/F^::AlbCre* mice compared to the other two genotypes (Figure 2B,C). At 4 W, transcript levels of these genes were generally higher in the *Keap1^F/F^::Rbpj^F/F^::AlbCre* and *Nrf2^F/F^::Keap1^F/F^::Rbpj^F/F^::AlbCre* mice compared to *Rbpj^F/F^::AlbCre* mice (Figure 4B).

#### 2.3.3. Effect on Bile Acid Synthesis-Related Gene Transcripts

CYP7A1 is the rate-limiting enzyme in rodents and humans [33] for the synthesis of bile acids from cholesterol. ABCB11, also known as the bile salt export pump, is the efflux protein for bile acids synthesized in hepatocytes for export into bile. Importantly, *ABCB11* has been associated with type 2 Progressive Familial Intrahepatic Cholestasis, which is a severe human genetic disease [34]. These two transcripts were examined at 3 W and 4 W of age. Interestingly, *Cyp7A1* transcripts in *Keap1^F/F^::Rbpj^F/F^::AlbCre* mice were lower by more than half compared to *Rbpj^F/F^::AlbCre* and *Nrf2^F/F^::Keap1^F/F^::Rbpj^F/F^::AlbCre* mice at 3 W. However, at 4 W, all three genotypes exhibited similar expression levels. *Abcb11* transcripts were reduced by 75% in *Keap1^F/F^::Rbpj^F/F^::AlbCre* mice, compared to *Rbpj^F/F^::AlbCre* mice (Figure 4C). Meanwhile, at 4 W, *Abcb11* transcripts were expressed to the same levels as seen in the case of *Cyp7A* transcripts (Figure 4C). NR0B2, also referred to as SHP (nuclear receptor small heterodimer partner), is known as a negative regulator for *Cyp7A1* expression responding to bile acid synthesis [35]. Consequently, it contributes to maintaining cholesterol and bile acid balance in the liver within the FXR gene expression system. *Nr0b2* transcript levels were lower in *Keap1^F/F^::Rbpj^F/F^::AlbCre* and especially *Nrf2^FF^::Keap1^F/F^::Rbpj^F/F^::AlbCre* mice compared to *Rbpj^F/F^::AlbCre* mice at 3 W. At 4 W, *Nr0b2* transcripts in *Nrf2^F/F^::Keap1^F/F^::Rbpj^F/F^::AlbCre* mice were almost double that of *Rbpj^F/F^::AlbCre* mice (Figure 4D).

### 2.4. Protein Level Alterations of Hepatic De Novo Lipid Synthesis-Related Gene Expression during Early Development among Key Genotypes

The results of the fatty acid- and triglyceride-related gene expression analyses and histochemical observations of oil-Red-O staining patterns at 3 W indicated disturbances in the cholesterol and subsequent bile acid synthesis/efflux pathways, which were possible primary responses. The cholesterol synthesis-related transcriptional response in *Rbpj^F/F^::AlbCre*, *Keap1^F/F^::Rbpj^F/F^::AlbCre* and *Nrf2^F/F^::Keap1^F/F^::Rbpj^F/F^::AlbCre* mice seemed to arise from a phase shift against hepatic cholestasis damage. Therefore, the amounts of representative functional proteins in the cholesterol and subsequent bile acid synthesis/efflux pathways were examined in 3 W- and 4 W-old mice.

**Figure 5:**
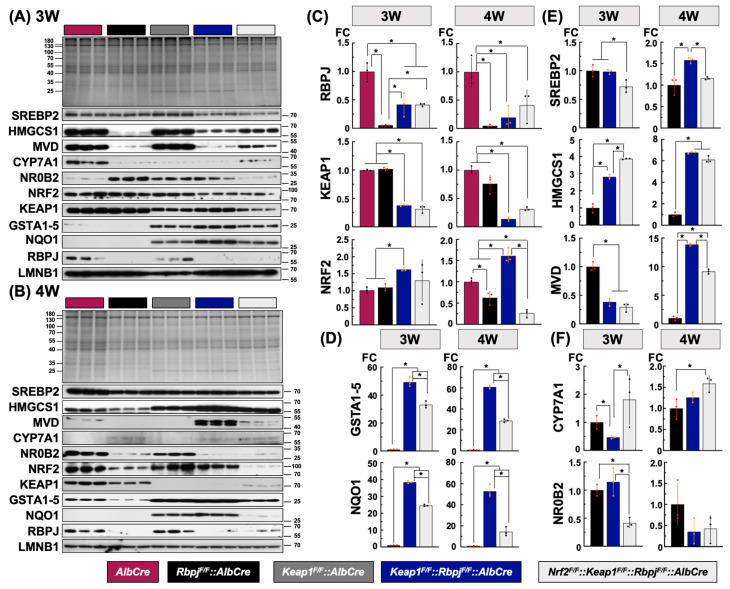
Immunoblotting analyses of putative ARE-regulated (NRF2) gene products in metabolic pathways affecting cholesterol–bile acid flux in 3 W- (**A**) and 4 W-old (**B**) mice. Liver extracts from 3 individual mice for each genotype were examined; quantification was performed by normalizing with LMNB1 expression. Deletion target gene products (**C**), representative NRF2 target genes (**D**), cholesterol synthesis-related gene products (**E**) and bile acid pathway-related gene products (**F**) are presented. The Y axis indicates the level of fold change (FC), where gene expression in *AlbCre* mice was set as 1 in (**C**), and for *Rbpj^F/F^*::*AlbCre* mice, it was set as 1 in (**D**–**F**). N = 3. * *p* < 0.05 by Tukey’s (highlighted by red or orange datapoints).

To further characterize the mice in this study that used flanking-LoxP targeting of *Keap1*, *Rbpjκ* and *Nrf2*, the floxed allele gene products were examined by immuno-blotting analyses (Figure 5A–C). In the case of RBPJκ especially in the 3 W liver, *Keap1^F/F^::Rbpj^F/F^::AlbCre* and *Nrf2^F/F^::Keap1^F/F^::Rbpj^F/F^::AlbCre* mice expressed 10 times higher levels than in *Rbpj^F/F^::AlbCre* mice, but KEAP1 showed no effects between *Keap1^F/F^::Rbpj^F/F^::AlbCre* and *Nrf2^F/F^::Keap1^F/F^::Rbpj^F/F^::AlbCre* mice. However, NRF2 was influenced by unexpectedly larger changes from the same comparisons. NRF2 protein in the *Nrf2^F/F^::Keap1^F/F^::Rbpj^F/F^::AlbCre* mouse liver was detected at a similarly higher NRF2 expression together with representative NRF2 target gene products, NQO1 and GSTAs, as in *Keap1^F/F^::Rbpj^F/F^::AlbCre* at 3 W (Figure 5D). However, in the 4 W liver, all Flox P target gene proteins fell to less than 25% of the amount seen in *AlbCre* mouse (Figure 5D). These results demonstrate a reasonable knockout effect, given that it only reflects the hepatoblast-derived cell population in the liver. Furthermore, the *Keap1^F/F^* mouse demonstrated constitutively higher NRF2 expression even under basal level conditions, due to the hypomorphic *Keap1* gene expression evoked by the insertional position of the LoxP element. Therefore, hepatic NRF2 in *Nrf2^F/F^::Keap1^F/F^::Rbpj^F/F^::AlbCre* mice, before functional Cre expression, might exhibit higher levels as in in *Keap1^F/F^::Rbpj^F/F^::AlbCre* mice. Even though genetic excision was successfully detected (Supplemental Figure S6), there was still the possibility that the remaining NRF2 in the hepatocytes of *Nrf2^F/F^::Keap1^F/F^::Rbpj^F/F^::AlbCre* mice could function in the livers of *Keap1^F/F^::Rbpj^F/F^::AlbCre* mice at 3 W under cholestasis conditions.

#### 2.4.1. Protein Expression Related to Hepatic Cholesterol Synthesis among the Cholestasis Genotypes

SREBP2 was detected as an active transcription factor in a 70kDa band [36] in the immunoblot analysis (Figure 5A,B). This band was detected in the livers of all genotyped mice (Figure 5A,B,E). Regardless of whether mice developed cholestasis or not, or the timepoints after birth, activated SREBP2 amounts were similar with only small changes among genotypes. Therefore, higher hepatic cholesterol levels in the cholestatic livers of *Rbpj^F/F^::AlbCre* and *Keap1^F/F^::Rbpj^F/F^::AlbCre* mice were considered not to be associated with SREBP2 actions. This view is consistent with the observation of equal levels of canonical SREBP2 target gene transcripts such as *Hmgcs1*, *Hmgcr*, *Mvd* and *Ldlr* between *Rbpj^F/F^::AlbCre* and *Keap1^F/F^::Rbpj^F/F^::AlbCre* mice (Figure 4B, 3 W). HMGCS1 was reduced in expression in livers exhibiting cholestasis based upon comparisons of *AlbCre* and *Rbpj^F/F^::AlbCre* or *Keap1^F/F^::Rbpj^F/F^::AlbCre* mice (Figure 5A). Perhaps the RBPJκ and NICD complex is involved in regulating *Hmgcs*1 expression. Meanwhile, as seen in comparisons of cholestatic livers in *Rbpj^F/F^::AlbCre* and *Keap1^F/F^::Rbpj^F/F^::AlbCre* mice, wherein *Keap1^F/F^::Rbpj^F/F^::AlbCre* mice had the highest cholesterol levels in both the plasma or liver, ~3-fold higher *Hmgcs1* expression was detected as in these mice at 3 W. This elevated expression pattern became substantially larger at 4 W. However, *Nrf2^F/F^::Keap1^F/F^::Rbpj^F/F^::AlbCre* livers at 4 W showed similarly high levels as in *Keap1^F/F^::Rbpj^F/F^::AlbCre* mice (Figure 5E). Probably, the direct NRF2 contributions for enhanced *Hmgcs1* expression in cholestasis livers are small, but there might be a KEAP1-dependent signaling effect.

*Mvd* (mevalonate decarboxylase) is a putative NRF2 target gene [37] that contains multiple ARE sequences on its proximal promoter region (to ~2 kb upstream from the transcription initiation site). MVD is also known as the rate-limiting enzyme in the mevalonate pathway [38]. It catalyzes mevalonate-5-diphosphate early in the process of cholesterol biosynthesis (acetoacetyl CoA to mevalonate) to isopentenyl diphosphate, which is essential for poly-isoprenoid synthesis [39]. These products are also involved in the positive regulation of cell proliferation. In the case of *Mvd* expression, its protein levels reflected transcript levels at both 3 and 4 W (Figure 4B). At 3 W, hepatic *Mvd* expressions in genotypes developing cholestasis were all lower than seen in control *AlbCre* mice. MVD in the livers of *Keap1^F/F^::Rbpj^F/F^::AlbCre* or *Nrf2^F/F^::Keap1^F/F^::Rbpj^F/F^::AlbCre* mice were less than 50% of the *Rbpj^F/F^::AlbCre* mice. In 4 W livers, MVD amounts changed dramatically. Remarkably higher MVD levels were expressed in livers exhibiting sustained cholestasis based upon the comparisons of *Rbpj^F/F^::AlbCre*, *Keap1^F/F^::Rbpj^F/F^::AlbCre* and *Nrf2^F/F^::Keap1^F/F^::Rbpj^F/F^::AlbCre* mice. Additionally, the comparison of MVD in the *Keap1^F/F^::Rbpj^F/F^::AlbCre* and *Nrf2^F/F^::Keap1^F/F^::Rbpj^F/F^::AlbCre* mice showed the possibility of involvement of NRF2 signaling due to the reduction in hepatic MVD levels in *Nrf2^F/F^::Keap1^F/F^::Rbpj^F/F^::AlbCre* mice (Figure 5E).

#### 2.4.2. Protein Expression Related to Hepatic Bile Acid Synthesis among the Cholestasis Genotypes

CYP7A1, which is reported to exhibit a liver-specific expression profile, functions to catalyze the first and rate-limiting step for bile acid biosynthesis from cholesterol [40]. Transcript expression patterns were reflected in the amounts of the protein measured in the different mouse genotypes. There were remarkably lower levels in the cholestasis livers at 3 W (Figure 5A, Supplemental Figure S9). CYP7A1, detected in the liver of the *Keap1^F/F^::Rbpj^F/F^::AlbCre* mouse, was lowest amongst the comparisons of mice damaged by cholestasis, being found in less than 50% of the *Rbpj^F/F^::AlbCre* mice. Interestingly, CYP7A1 in *Nrf2^F/F^::Keap1^F/F^::Rbpj^F/F^::AlbCre* mice was detected as the highest, with concentrations more than three times those seen in *Keap1^F/F^::Rbpj^F/F^::AlbCre* mice. Thus, there is a possibility of a suppressive effect of NRF2 signaling on CYP7A1 gene expression in the 3 W liver. From the 4 W results, the expression level became ~3-fold higher in cholestatic livers than in the non-damaged livers of the *AlbCre* and *Keap1^F/F^::AlbCre* mice (Supplemental Figure S9). As the negative regulator of *Cyp7A1*, NR0B2 was expressed to ~3-fold higher levels in the livers that exhibited lower CYP7A1 expression at 3 W but there was no difference between *Rbpj^F/F^::AlbCre* and *Keap1^F/F^::Rbpj^F/F^::AlbCre* mice (Figure 5F, Supplemental Figure S9), except for *Nrf2^F/F^::Keap1^F/F^::Rbpj^F/F^::AlbCre* mice. At 4 W, conversely, in the higher CYP7A1 expression genotypes, NR0B2 was reduced to lower levels than in the control *AlbCre* and *Keap1^F/F^::AlbCre* mice, which did not exhibit cholestatic liver damage (Supplemental Figure S9). There was no statistical difference in protein levels among the livers developing cholestasis (Figure 5F).

## 3. Discussion

*Rbpj^F/F^::AlbCre* mice develop cholestasis due to hypomorphic intrahepatic bile duct development in the early postnatal period [10,30]. However, the livers of *Rbpj^F/F^::AlbCre* mice are able to compensate by constructing intrahepatic bile ducts independent of NOTCH signaling through the process of cellular plasticity [10]. Specifically, SOX9, which is a biliary lineage marker and transcription factor—and debated as an NRF2 target gene product [41]—is reported as a key factor acquired in periportal as well as interlobular hepatocytes to generate an hepato-cholangio intermediate cell phenotype [42]. At the initiation of the present research, we estimated that a *Keap1^F/F^::Rbpj^F/F^::AlbCre* mouse could block the development of or improve recovery from the cholestatic symptoms seen in *Rbpj^F/F^::AlbCre* mice based on the gene ontology of KEAP1-NRF2 signaling contributions towards cellular defense mechanisms and metabolism [43,44,45]. Indeed, it has been reported that the cytoprotective genes against oxidative stress, detoxification enzyme genes or phase 3 membrane efflux transporter genes, such as the multidrug resistance-associated protein (MRP) 2, MRP3 and MRP4, were up-regulated by the KEAP1-NRF2 system and ameliorated bile acid-induced cholestatic liver injury in mice [46,47]. Contrary to our expectation, the symptoms of cholestasis evoked by depleting NOTCH signaling were enhanced by NRF2 signaling in the livers of *Keap1^F/F^::Rbpj^F/F^::AlbCre* mice and were extended through the growth stage until at least 9 W. Consequently, remarkable hepatomegaly and hypercholesterolemia accompanied by hepatic damage were amplified and sustained (Figure 1 and Figure 2). Therefore, it was important to understand what gene expression changes occurred and how altered NRF2 signaling contributed to abnormal lipid metabolism in the liver between *Rbpj^F/F^::AlbCre* and *Keap1^F/F^::Rbpj^F/F^::AlbCre* mice. Because both the control *AlbCre* and *Keap1^F/F^::AlbCre* mice did not express any cholestatic symptoms, it was determined that the key differential changes in gene expression focused on lipid metabolism could be evaluated effectively from comparisons of hepatic expression levels in *Rbpj^F/F^::AlbCre*, *Keap1^F/F^::Rbpj^F/F^::AlbCre* and *Nrf2^F/F^::Keap1^F/F^::Rbpj^F/F^::AlbCre* mice.

### 3.1. Fatty Acids and Triglycerides Synthetic Pathway

Serum ALT and hepatic triglyceride levels at 3 W were not different among *Rbpj^F/F^::AlbCre*, *Keap1^F/F^::Rbpj^F/F^::AlbCre* and *Nrf2^F/F^::Keap1^F/F^::Rbpj^F/F^::AlbCre* mice. Altered expression of fatty acid and triglyceride synthesis-related genes may not contribute much to the process of cholestasis. However, transcript levels of de novo lipogenesis genes such as *Acc1*, *Fasn* and *Scd1* declined in *Rbpj^F/F^::AlbCre* and *Keap1^F/F^::Rbpj^F/F^::AlbCre* livers despite small changes in *Srebp1c* mRNA expression. Perhaps there is a repressive effect of activation of NRF2 signaling due to cholestatic damage of the liver independent of the SREBP1c system. In fact, given the reductive effect on hepatic triglycerides from 4 W to 9 W, this process appears to be influenced by NRF2 signaling in agreement with other studies [48]. Namely, inhibiting lipogenesis through NRF2 signaling may give rise to sustained hypercholesterolemia and resultant hepatic damage.

### 3.2. Cholesterol Synthetic Pathway

Generally, SREBP2 contributes to the expression of cholesterol synthesis-related genes [32,36]. The hepatic expression profile of *Srebp2* transcripts and SREBP2 protein observed at 3 W was quite similar between *Rbpj^F/F^::AlbCre* and *Keap1^F/F^::Rbpj^F/F^::AlbCre* mice, despite significantly higher-levels of cholesterol in the liver and plasma than in the homeostatic condition of *AlbCre* mice. Since the expression of SREBP2 direct target genes, such as *Hmgcs1*, *Hmgcr*, *Mvd* and *Ldlr* transcripts, was all lower than in *AlbCre* mice, a negative feedback response against excess cholesterol in the liver may have happened quickly at the transcriptional level. Given that amounts of activated SREBP2 protein were quite similar, transactivation through SREBP2 signaling seems unlikely. Altered NOTCH or NRF2 signaling might affect cholesterol synthesis; however, the accumulation of hepatic cholesterol was not associated with the activation of the biosynthesis pathway under the conditions of cholestasis at 3 W. In 4 W-old post-weaning *Rbpj^F/F^::AlbCre* mice, hepatic transcript levels of cholesterogenesis genes were upregulated remarkably and accompanied by *Srebp2* transcripts compared to mice of the *Keap1^F/F^::Rbpj^F/F^::AlbCre* and *Nrf2^F/F^::Keap1^F/F^::Rbpj^F/F^::AlbCre* genotypes. Perhaps KEAP1-dependent and NRF2-independent gene expression systems are involved. Because there is no detectable change in the quantity of activated SREBP2, the potential of NRF1 to be an effector is conceivable, given the multiple putative ARE sequences on the promoter regions of the examined SREBP2 target genes by in silico analyses and possible association to KEAP1 through the NRF1-Neh2 domain [49,50]. It has been reported that NRF1 functioned as a sensor to cholesterol in the endoplasmic reticulum, depending upon feeding a diet supplemented with cholesterol and cholate. Mice with hepatic NRF1 deficiency excrete less and accumulate more cholesterol in liver [51]. Some of genes have been identified by the comparison of RNAseq and Chip combination analyses using the livers of *Nrf1^F/F^*, *Nrf2^F/F^* and *Nrf1^F/F^:: Nrf2^F/F^* mice infected with a liver-targeting adeno-associated virus expressing Cre recombinase via thyroxine binding globulin promoter [52]. A complementary gene regulatory machinery by NRF1 and NRF2 may modulate cholesterol metabolism in mice. While it was a feature of *Rbpj^F/F^::AlbCre* mice, there was no elevation of gene transcripts for hepatic cholesterol synthesis, such as *Hmgcs1* and *Hmgcr*, but there was for the cholesterol uptake gene *Ldlr* and mevalonate pathway enzyme *Mvd*. Levels of these genes were corelated to protein quantities. Perhaps both the quick reduction in cholesterol biosynthesis and activation of a recycling pathway for circulating cholesterol contribute to the resolution of the elevated cholesterol levels observed by 4 W in the *Rbpj^F/F^::AlbCre* mouse liver.

### 3.3. Bile Acid Synthetic Pathway

Within the bile acid synthetic pathway, *Cyp7A1* and *Abcb11* expression were lower than in the *AlbCre* mouse. Both transcript and protein levels of *Cyp7A1* were reduced in the cholestatic liver at 3 W. From in silico analyses, the core sequences for the RBPJ-binding element [53] and ARE were found in the proximal promoter region of murine *Cyp7A1*. In the case of human *CYP7A1* expression, NRF2 was reported to be crucial for the downregulation of CYP7A1 in HepG2 cells [54].

Interestingly, Cyp7A1 expression in the *Keap1^F/F^::Rbpj^F/F^::AlbCre* liver was less than half of that in the *Rbpj^F/F^::AlbCre* liver. Simultaneously, NR0B2 [55], which is known as a crucial suppressor for *CYP7A1* and *Cyp8B1* gene expression with LXRα or LRH1 [35], exhibited upregulated protein levels in both *Rbpj^F/F^::AlbCre* and *Keap1^F/F^::Rbpj^F/F^::AlbCre* livers at 3 W. Due to the lack of correlation with levels of *Nr0b2* transcripts, altered NOTCH signaling or NRF2 signaling could provide higher stability for the NR0B2 protein. Given the function of CYP7A1 as the rate-limiting enzyme in bile acid synthesis [40], it is likely that cholesterol catabolism might be downregulated. Even small MAFG, which is a direct NRF2 target gene and heterodimeric partner with NRF2 for binding to AREs in both humans and mice [56], has been reported to exert a direct repressor function for *Cyp8B1* expression through a small MAF recognition element on its promoter [57]. Furthermore, microarray results of differential hepatic gene expression between 4 W-old *Rbpj^F/F^::AlfpCre* and its control mouse (*Rbpj^F/F^*) revealed that *Cyp7B1* expression was considerably lower in the *Rbpj^F/F^::AlfpCre* liver [10]. These results indicate that cholesterol could not be metabolized into bile acids more efficiently in *Keap1^F/F^::Rbpj^F/F^::AlbCre* than *Rbpj^F/F^::AlbCre* or the wild-type liver.

**Figure 6:**
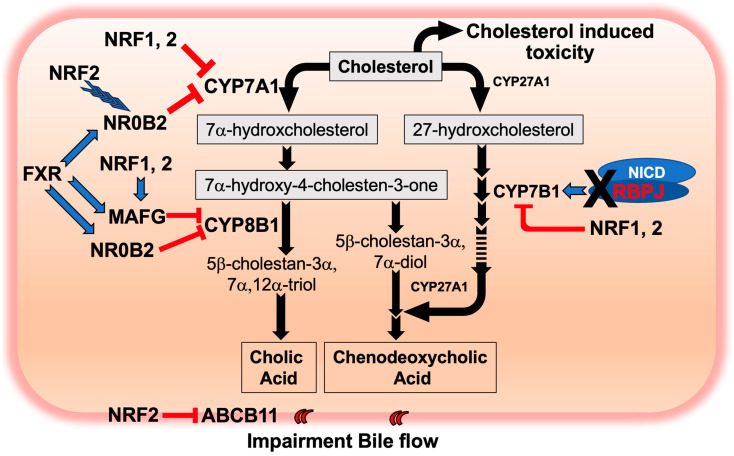
Possible gene expression changes from enhanced NRF2 signaling and depleted NOTCH signaling in the bile acid synthetic pathway from cholesterol in *Keap1^F/F^::Rbpj^F/F^::AlbCre* liver at 3 W of age. Blue arrows indicate activation of gene expression and red arrows ending in perpendicular red lines (I) indicate gene repression. Blue arrowheads indicate indirect activation such as effects on protein level stability.

Apparently, intrahepatic cellular cholesterol might exceed a permissible level for cell survival. It was reported that excess cholesterol leads to a collapse of membrane function and causes the formation of intrahepatocyte crystals that enhance oxidative damage, organelle dysfunction, inflammation and fibrosis [58,59,60,61,62]. Despite the response of the liver to this stress by eliminating cholesterol via hepato-biliary transport, either by direct excretion into bile or conversion to bile acids for excretion into the bile duct, the outcome is worse in *Keap1^F/F^::Rbpj^F/F^::AlbCre* mice. A reduction in gene expression (~30% of *Abcb11* transcripts in *Rbpj^F/F^::AlbCre* mouse liver) required for the formation of the bile acid secretory pump was measured during the pre-weaning stage. Bile acid accumulation probably elicits toxic detergent effects and raises ROS levels in hepatocytes. After weaning, cholesterol catabolism and bile acid efflux might turn towards recovery in the *Rbpj^F/F^::AlbCre* mouse liver, but apparently cholesterol synthetic and recycling pathways might occur to activate the mevalonate pathway in the *Keap1^F/F^::Rbpj^F/F^::AlbCre* liver in a *Keap1*-dependent, *Nrf2*-independent manner. These metabolic effects and the interplay with the ARE-NRF2 signaling pathway are depicted in Figure 6.

### 3.4. How Does Depletion of NOTCH and Enhanced NRF2 Signaling Exacerbate Hyperlipidemia?

NRF2 and NOTCH signaling are known to influence pleiotropic responses affecting cellular and organismal homeostasis. The foundational observation for this study was that the depletion of NOTCH signaling due to hepatoblast lineage-specific loss of *Rbpj* resulted in cholestasis in the postnatal liver, which was likely triggered by bile duct dysplasia [10,29,30]. As the co-deletion of *Keap1* and *Rbpj* genes evolved from the expression of the albumin Cre-Lox-P system during the pre-weaning stage, cholesterol metabolism markedly declined due to the suppression of *Cyp7A1* gene expression. The depletion of NOTCH signaling upregulated transcription of the transcription factor NR0B2, which, in turn, repressed *Cyp7A1* expression. NRF2 signaling could provide NR0B2 stability and repress *Cyp7A1* gene expression through collaboration with NR0B2 or perhaps independently. As a result, cholesterol accumulates and induces toxicity to hepatocytes. Moreover, *Keap1*-deletion might contribute to the repression of *ABCB11* gene expression initially in an NRF2-independent manner at 3 W but may evolve to an NRF2-dependent manner by 4 W. How this transition might occur is unknown. Nonetheless, the result is enhanced bile acid accumulation, leading to elevated ROS levels in cells and toxicity.

These events disturb the elimination of cholesterol and the coordinated metabolism of bile acids that normally maintain a homeostatic rate of elimination during the pre-weaning stage of postnatal development. While the NOTCH signaling pathway has been linked to diseases like biliary atresia and Alagille syndrome—two rare cholestatic diseases during early childhood—the toxicity-amplifying interaction with the NRF2 signaling pathway was unanticipated. Despite the well-described actions of NRF2 on cell survival processes, including the attenuation of oxidative stress, enhancement of NRF2 signaling by deletion of its repressor, *Keap1* failed to impede, and in fact exacerbated, the extent and duration of cholestasis evoked by *Rbpjκ* depletion from hepatoblast lineage cells. Further study is needed to understand the consequences of cross-talk between sometimes complementary and sometimes antagonistic signaling pathways such as NOTCH and NRF2.

## 4. Materials and Methods

### 4.1. Animals

Mice were maintained at 22 °C, 50% humidity with a 12 h light/dark cycle and ad libitum access to water and food (PicoLab^®^ Rodent Diet 20 5053 irradiated diet, LabDiet, Arden Hills, MN, USA). All mice were of the albino C57BL/6J background (B6(Cg)-Tyrc-2J/J) (Jackson Laboratories, Bar Harbor, ME). Keap1 floxed (*Keap1^F/F^*) [22] and Nrf2 null (*Nrf2^−/−^*) [18] mice were kindly provided by Prof. Masayuki Yamamoto, Division of Medical Biochemistry, Tohoku University School of Medicine, Sendai, Japan and Nrf2 floxed mice (*Nrf2^F/F^*) [63] were provided by Prof. Shyam Biswal, Johns Hopkins Bloomberg School of Public Health, Baltimore, MD, USA. B6; Cg-Tg (AlbCre)21Mgn/J (AlbCre) [64] and Rbpjκ floxed mice, (*Rbpj^F/F^*) [65] mice were obtained from Jackson Laboratories (Bar Harbor, ME, USA) and RIKEN BioResource Center (Tsukuba, Japan), respectively. *Keap1^F/F^*, *Nrf2^F/F^* or *Rbpj^F/F^::AlbCre* mice were generated by crossing *Keap1^F/F^*, *Nrf2^F/F^* or *Rbpj^F/F^* mice with *AlbCre*, respectively. *Keap1^F/F^::Rbpj^F/F^::AlbCre* were established by consecutive mating of *Keap1^F/+^::Rbpj^F/+^::AlbCre*, which were descendants from *Keap1^F/F^::AlbCre* and *Rbpj^F/F^* pairs. *Nrf2^F/F^::Keap1^F/F^::Rbpj^F/F^::AlbCre* mice or *Nrf2^−/−^::Keap1^F/F^::Rbpj^F/F^::AlbCre* mice were established by consecutive mating from *Nrf2^F/+^::Keap1^F/+^::Rbpj^F/+^::AlbCre* or *Nrf2^+/−^::Keap1^F/+^::Rbpj^F/+^::AlbCre* mice, utilizing *Nrf2^F/F^::AlbCre* or *Nrf2^−/−^::AlbCre* and *Keap1^F/F^::Rbpj^F/F^* pairs. Mice were maintained by pairing males bearing AlbCre-transgenic alleles with non-Cre gene carrier females. All mice were evaluated by standard PCR-genotyping. The PCR conditions and primers, and locations used for the genotyping of each line, are described in Supplemental Figures S7 and S8, Tables S1–S3 and previous work [28,66]. All mouse experiments were performed at the Fred Hutchinson Cancer Center and were approved by the Institutional Animal Care and Use Committee (protocol #51042).

### 4.2. Blood Collection for Biochemical Analyses and Dissection of Liver

Dissection of mice was performed entirely under anesthesia using 3% isoflurane with oxygen delivered at 0.5 L/min (Piramal Critical Care, Bethlehem, PA, USA) with a vaporizer (Surgivet model 100, Smiths Medical North America, Waukesha, WI, USA). Mice were cut with a Y-shaped incision along the abdominal surface using sterilized surgical tools; intestines were gently moved to the right side. Blood was collected through the inferior vena cava using a heparinized 25G needle with 1 mL syringe. Blood was kept on ice until centrifugation at 3000× *g* for 30 min at 4 °C for plasma isolation. The plasma biochemical analyses were conducted by commercial laboratories (Zoetis Reference Laboratories, Mukilteo, WA, USA or Moichor Reference Labs, San Francisco, CA, USA). Following blood collection, the entire liver was dissected and weighed. One-third of both the center and left lobes were cut for histological analyses, and the remaining liver was flash frozen and kept at −80 °C prior to molecular and biochemical analyses. Mice were euthanized by cervical dislocation. After confirming that the heart had stopped beating and breathing was no longer taking place, 1 mm of each tail was cut and genomic DNA isolated for the confirmation of genotype. In the case of stereomicroscopic observations, immediately after isolating the liver, it was soaked in ice-cold Hank’s buffer and assessed using the stereoscope SZX12 (Olympus, Waltham, MA, USA).

### 4.3. Histology

Mouse livers were isolated and fixed in 4% paraformaldehyde (PFA), then embedded in paraffin and sectioned. Sections were then deparaffinized using heat and xylene. Tissues were rehydrated using reducing concentrations of ethanol, then finally in water. Slides were stained with eosin and counterstained with hematoxylin (Thermo Fisher Scientific, Waltham, MA, USA). The sections were dehydrated using increasing concentrations of ethanol, cleared using xylene, and finally mounted using Paramount mounting medium (Thermo Fisher Scientific, Fisher Scientific, Waltham, MA, USA). Images were viewed and recorded on a Nikon Eclipse E800 Core microscope (Nikon, Melville, NY, USA). Optimal cutting temperature (OCT) compound blocks were prepared by embedding livers in OCT compound (Sakura Finetek USA, Torrance, CA, USA). Slides were fixed with 4% PFA at 4 °C for 10 min, then stained with oil Red O solution for 120 min at room temperature. Liver sections were counterstained with hematoxylin (Carolina Biological Supply Company, Burlington, NC, USA).

### 4.4. Isolation and Purification of Total RNA and RT-PCR

Livers, flash frozen in liquid N_2_ (stored at −80 °C until use), were homogenized in TRIzol (Thermo Fisher Scientific, Waltham, MA, USA). Extracted total RNA was purified by using the RNeasy mini-kit (Qiagen, Germantown, MD, USA). RNA integrity was confirmed by electrophoresis before the reverse transcriptase (RT) reaction. RNA quantification was performed by spectrophotometry at 260 nm. Genes of interest were analyzed by SYBR green real-time quantitative RT-PCR (qPCR). cDNA was synthesized by using the qScript system (Quanta Biosciences, Beverly, MA, USA). Realtime PCR was performed by QuantStudio 7 (Applied Biosystems, Waltham, MA, USA) with TaqMan Fast Advanced Master Mix (Applied Biosystems, Waltham, MA, USA). The primers are shown in Supplementary Table S4 [67,68]. Expression levels of each gene were normalized to 18S rRNA and calculated as relative to control.

### 4.5. Hepatic Triglyceride and Cholesterol Assay

Hepatic lipid content of mice was assayed using biochemical assays. Approximately 100 mg (triglyceride assay) or 10 mg (cholesterol assay) of flash frozen livers was extracted and resolubilized in aqueous media and quantified using kits: Cayman 10010303 triglycerides assay kit (Cayman, Ann Arbor, MI, USA) or Cholesterol Assay Kit STA-384 (Cell BioLabs, San Diego, CA, USA). Spectrophotometric measurement was performed using a SpectraMax M5 plate reader (Molecular Devices, San Jose, CA, USA).

### 4.6. Immune Blotting Analyses

Proper-size-cut tissues were homogenized in RIPA-I buffer, which contained a protease inhibitor cocktail (Roche, So. San Francisco, CA, USA). This solution was assayed for protein concentration by the Bio-Rad protein assay (Bio-Rad, Hercules, CA, USA) using bovine serum albumin to generate a standard curve. A 10 mg/mL RIPA-I solution is an ideal concentration for the immune-blotting analyses. An equal volume of 2× SDS sample buffer was added, and the samples were denatured by boiling for 5 min. Samples were applied onto SDS-PAGE gels and transferred onto an Immobilon polyvinylidene difluoride membrane (Millipore, Burlington, MA, USA). The membranes were blocked with Tris-buffered saline with 0.05% Tween 20 and 5% skim milk (Difco, Tucker, GA, USA) and then treated with each primary antibody listed in Supplemental Table S5. The preparative membranes were reacted with appropriate secondary antibodies conjugated to horseradish peroxidase (Invitrogen, Waltham, MA, USA). The immunocomplexes were visualized with ECL (PerkinElmer, Waltham, MA, USA). Detected band intensities were quantified by Image J and normalized by the nuclear Lamin B1 band.

### 4.7. Statistical Analysis

GraphPad Prism 9.5.1 (528) was used for statistical analyses of data sets. Quantitative data are presented as mean ± SD (Figure 1, Figure 2 and Figure 5), mean ± SE (Figure 3 and Figure 4). For more than two groups, one-way ANOVA was used followed by Tukey’s test, as described in the relevant figure legends.

## Data Availability

All data presented are contained within the manuscript/Supplementary Materials.

## Supplementary Materials

The following supporting information can be downloaded at: https://www.mdpi.com/article/10.3390/ijms25094712/s1.

## References

[1] Musso G., Gambino R., Cassader M. (2013). Cholesterol metabolism and the pathogenesis of non-alcoholic steatohepatitis. Prog. Lipid. Res..

[2] Gilbert M.A., Bauer R.C., Rajagopalan R., Grochowski C.M., Chao G., McEldrew D., Nassur J.A., Rand E.B., Krock B.L., Kamath B.M. (2019). Alagille syndrome mutation update: Comprehensive overview of JAG1 and NOTCH2 mutation frequencies and insight into missense variant classification. Hum. Mutat..

[3] Deutsch G.H., Sokol R.J., Stathos T.H., Knisely A.S. (2001). Proliferation to paucity: Evolution of bile duct abnormalities in a case of Alagille syndrome. Pediatr. Dev. Pathol..

[4] Furukawa T., Kawaichi M., Matsunami N., Ryo H., Nishida Y., Honjo T. (1991). The Drosophila RBP-J kappa gene encodes the binding protein for the immunoglobulin J kappa recombination signal sequence. J. Biol. Chem..

[5] Hamaguchi Y., Yamamoto Y., Iwanari H., Maruyama S., Furukawa T., Matsunami N., Honjo T. (1992). Biochemical and immunological characterization of the DNA binding protein (RBP-J kappa) to mouse J kappa recombination signal sequence. J. Biochem..

[6] Artavanis-Tsakonas S., Matsuno K., Fortini M.E. (1995). Notch signaling. Science.

[7] Weisend C.M., Kundert J.A., Suvorova E.S., Prigge J.R., Schmidt E.E. (2009). Cre activity in fetal albCre mouse hepatocytes: Utility for developmental studies. Genesis.

[8] Postic C., Magnuson M.A. (2000). DNA excision in liver by an albumin-Cre transgene occurs progressively with age. Genesis.

[9] Kellendonk C., Opherk C., Anlag K., Schütz G., Tronche F. (2000). Hepatocyte-specific expression of Cre recombinase. Genesis.

[10] Tharehalli U., Svinarenko M., Kraus J.M., Kuhlwein S.D., Szekely R., Kiesle U., Scheffold A., Barth T.F.E., Kleger A., Schirmbeck R. (2018). YAP Activation Drives Liver Regeneration after Cholestatic Damage Induced by Rbpj Deletion. Int. J. Mol. Sci..

[11] Dědič T., Jirsa M., Keil R., Rygl M., Šnajdauf J., Kotalová R. (2015). Alagille Syndrome Mimicking Biliary Atresia in Early Infancy. PLoS ONE.

[12] Davenport M. (2012). Biliary atresia: Clinical aspects. Semin Pediatr. Surg..

[13] Nakajima H., Tsuma Y., Fukuhara S., Kodo K. (2022). A case of Infantile Alagille Syndrome with severe dyslipidemia: NewiInsight into lipid metabolism and therapeutics. J. Endocr. Soc..

[14] Taylor S.A., Chen S.Y., Gadhvi G., Feng L., Gromer K.D., Abdala-Valencia H., Nam K., Dominguez S.T., Montgomery A.B., Reyfman P.A. (2021). Transcriptional profiling of pediatric cholestatic livers identifies three distinct macrophage populations. PLoS ONE.

[15] Vyas D., Baptista P.M., Brovold M., Moran E., Gaston B., Booth C., Samuel M., Atala A., Soker S. (2018). Self-assembled liver organoids recapitulate hepatobiliary organogenesis in vitro. Hepatology.

[16] Itoh K., Igarashi K., Hayashi N., Nishizawa M., Yamamoto M. (1995). Cloning and characterization of a novel erythroid cell-derived CNC family transcription factor heterodimerizing with the small Maf family proteins. Mol. Cell Biol..

[17] Singh A., Happel C., Manna S.K., Acquaah-Mensah G., Carrerero J., Kumar S., Nasipuri P., Krausz K.W., Wakabayashi N., Dewi R. (2013). Transcription factor NRF2 regulates miR-1 and miR-206 to drive tumorigenesis. J. Clin. Investig..

[18] Itoh K., Chiba T., Takahashi S., Ishii T., Igarashi K., Katoh Y., Oyake T., Hayashi N., Satoh K., Hatayama I. (1997). An Nrf2/small Maf heterodimer mediates the induction of phase II detoxifying enzyme genes through antioxidant response elements. Biochem. Biophys. Res. Commun..

[19] Yagishita Y., Chartoumpekis D.V., Kensler T.W., Wakabayashi N. (2023). NRF2 and the Moirai: Life and death decisions on cell fates. Antioxid. Redox Signal..

[20] Itoh K., Wakabayashi N., Katoh Y., Ishii T., Igarashi K., Engel J.D., Yamamoto M. (1999). Keap1 represses nuclear activation of antioxidant responsive elements by Nrf2 through binding to the amino-terminal Neh2 domain. Genes Dev..

[21] Kobayashi A., Kang M.I., Okawa H., Ohtsuji M., Zenke Y., Chiba T., Igarashi K., Yamamoto M. (2004). Oxidative stress sensor Keap1 functions as an adaptor for Cul3-based E3 ligase to regulate proteasomal degradation of Nrf2. Mol. Cell Biol..

[22] Okawa H., Motohashi H., Kobayashi A., Aburatani H., Kensler T.W., Yamamoto M. (2006). Hepatocyte-specific deletion of the keap1 gene activates Nrf2 and confers potent resistance against acute drug toxicity. Biochem. Biophys. Res. Commun..

[23] Wakabayashi N., Itoh K., Wakabayashi J., Motohashi H., Noda S., Takahashi S., Imakado S., Kotsuji T., Otsuka F., Roop D.R. (2003). Keap1-null mutation leads to postnatal lethality due to constitutive Nrf2 activation. Nat. Genet..

[24] Kwak M.K., Wakabayashi N., Itoh K., Motohashi H., Yamamoto M., Kensler T.W. (2003). Modulation of gene expression by cancer chemopreventive dithiolethiones through the Keap1-Nrf2 pathway. Identification of novel gene clusters for cell survival. J. Biol. Chem..

[25] Huang J., Tabbi-Anneni I., Gunda V., Wang L. (2010). Transcription factor Nrf2 regulates SHP and lipogenic gene expression in hepatic lipid metabolism. Am. J. Physiol. Gastrointest Liver Physiol..

[26] Zhang Y.K., Yeager R.L., Tanaka Y., Klaassen C.D. (2010). Enhanced expression of Nrf2 in mice attenuates the fatty liver produced by a methionine- and choline-deficient diet. Toxicol. Appl. Pharmacol..

[27] Chartoumpekis D.V., Ziros P.G., Psyrogiannis A.I., Papavassiliou A.G., Kyriazopoulou V.E., Sykiotis G.P., Habeos I.G. (2011). Nrf2 represses FGF21 during long-term high-fat diet-induced obesity in mice. Diabetes.

[28] Wakabayashi N., Skoko J.J., Chartoumpekis D.V., Kimura S., Slocum S.L., Noda K., Palliyaguru D.L., Fujimuro M., Boley P.A., Tanaka Y. (2014). Notch-Nrf2 axis: Regulation of Nrf2 gene expression and cytoprotection by notch signaling. Mol. Cell Biol..

[29] Sparks E.E., Huppert K.A., Brown M.A., Washington M.K., Huppert S.S. (2010). Notch signaling regulates formation of the three-dimensional architecture of intrahepatic bile ducts in mice. Hepatology.

[30] Sparks E.E., Perrien D.S., Huppert K.A., Peterson T.E., Huppert S.S. (2011). Defects in hepatic Notch signaling result in disruption of the communicating intrahepatic bile duct network in mice. Dis. Model Mech..

[31] Shimano H., Sato R. (2017). SREBP-regulated lipid metabolism: Convergent physiology-divergent pathophysiology. Nat. Rev. Endocrinol..

[32] Horton J.D., Goldstein J.L., Brown M.S. (2002). SREBPs: Activators of the complete program of cholesterol and fatty acid synthesis in the liver. J. Clin. Investig..

[33] Kang S., Davis R.A. (2000). Cholesterol and hepatic lipoprotein assembly and secretion. Biochim. Biophys. Acta.

[34] Jansen P.L., Strautnieks S.S., Jacquemin E., Hadchouel M., Sokal E.M., Hooiveld G.J., Koning J.H., De Jager-Krikken A., Kuipers F., Stellaard F. (1999). Hepatocanalicular bile salt export pump deficiency in patients with progressive familial intrahepatic cholestasis. Gastroenterology.

[35] Goodwin B., Jones S.A., Price R.R., Watson M.A., McKee D.D., Moore L.B., Galardi C., Wilson J.G., Lewis M.C., Roth M.E. (2000). A regulatory cascade of the nuclear receptors FXR, SHP-1, and LRH-1 represses bile acid biosynthesis. Mol. Cell.

[36] Edwards P.A., Tabor D., Kast H.R., Venkateswaran A. (2000). Regulation of gene expression by SREBP and SCAP. Biochim. Biophys. Acta.

[37] Wible R.S., Tran Q.T., Fathima S., Sutter C.H., Kensler T.W., Sutter T.R. (2018). Pharmacogenomics of Chemically Distinct Classes of Keap1-Nrf2 Activators Identify Common and Unique Gene, Protein, and Pathway Responses In Vivo. Mol. Pharmacol..

[38] Ramachandran C.K., Shah S.N. (1976). Decarboxylation of mevalonate pyrophosphate is one rate-limiting step in hepatic cholesterol synthesis in suckling and weaned rats. Biochem. Biophys. Res. Commun..

[39] Chen C.L., Paul L.N., Mermoud J.C., Steussy C.N., Stauffacher C.V. (2020). Visualizing the enzyme mechanism of mevalonate diphosphate decarboxylase. Nat. Commun..

[40] Chiang J.Y.L., Ferrell J.M. (2020). Up to date on cholesterol 7 alpha-hydroxylase (CYP7A1) in bile acid synthesis. Liver Res..

[41] Kubo Y., Beckmann R., Fragoulis A., Conrads C., Pavanram P., Nebelung S., Wolf M., Wruck C.J., Jahr H., Pufe T. (2022). Nrf2/ARE Signaling Directly Regulates SOX9 to Potentially Alter Age-Dependent Cartilage Degeneration. Antioxidants.

[42] Walter T.J., Vanderpool C., Cast A.E., Huppert S.S. (2014). Intrahepatic bile duct regeneration in mice does not require Hnf6 or Notch signaling through Rbpj. Am. J. Pathol..

[43] Aghagolzadeh P., Radpour R., Bachtler M., van Goor H., Smith E.R., Lister A., Odermatt A., Feelisch M., Pasch A. (2017). Hydrogen sulfide attenuates calcification of vascular smooth muscle cells via KEAP1/NRF2/NQO1 activation. Atherosclerosis.

[44] Brennan M.S., Patel H., Allaire N., Thai A., Cullen P., Ryan S., Lukashev M., Bista P., Huang R., Rhodes K.J. (2016). Pharmacodynamics of Dimethyl Fumarate Are Tissue Specific and Involve NRF2-Dependent and -Independent Mechanisms. Antioxid. Redox Signal..

[45] Chartoumpekis D.V., Ziros P.G., Zaravinos A., Iskrenova R.P., Psyrogiannis A.I., Kyriazopoulou V.E., Sykiotis G.P., Habeos I.G. (2013). Hepatic gene expression profiling in Nrf2 knockout mice after long-term high-fat diet-induced obesity. Oxid. Med. Cell Longev..

[46] Zhang Y., Lickteig A.J., Liu J., Csanaky I.L., Klaassen C.D. (2020). Effects of ablation and activation of Nrf2 on bile acid homeostasis in male mice. Toxicol. Appl. Pharmacol..

[47] Pang X., Tang C., Cao P., Zhou L., Chen X. (2021). Metabolic Activation of Retrorsine may Disrupt Bile Acid Homeostasis in Mice through the Nrf2 Pathway. Curr. Drug Metab..

[48] Hayes J.D., Dinkova-Kostova A.T. (2014). The Nrf2 regulatory network provides an interface between redox and intermediary metabolism. Trends Biochem. Sci..

[49] Johnsen O., Murphy P., Prydz H., Kolsto A.B. (1998). Interaction of the CNC-bZIP factor TCF11/LCR-F1/Nrf1 with MafG: Binding-site selection and regulation of transcription. Nucleic Acids Res..

[50] Hayes J.D., McMahon M. (2001). Molecular basis for the contribution of the antioxidant responsive element to cancer chemoprevention. Cancer Lett..

[51] Widenmaier S.B., Snyder N.A., Nguyen T.B., Arduini A., Lee G.Y., Arruda A.P., Saksi J., Bartelt A., Hotamisligil G.S. (2017). NRF1 Is an ER Membrane Sensor that Is Central to Cholesterol Homeostasis. Cell.

[52] Akl M.G., Li L., Baccetto R., Phanse S., Zhang Q., Trites M.J., McDonald S., Aoki H., Babu M., Widenmaier S.B. (2023). Complementary gene regulation by NRF1 and NRF2 protects against hepatic cholesterol overload. Cell Rep..

[53] Tun T., Hamaguchi Y., Matsunami N., Furukawa T., Honjo T., Kawaichi M. (1994). Recognition sequence of a highly conserved DNA binding protein RBP-J kappa. Nucleic Acids Res..

[54] Zhang J.M., Wang X.H., Hao L.H., Wang H., Zhang X.Y., Muhammad I., Qi Y., Li G.L., Sun X.Q. (2017). Nrf2 is crucial for the down-regulation of Cyp7a1 induced by arachidonic acid in Hepg2 cells. Environ. Toxicol. Pharmacol..

[55] Garruti G., Wang H.H., Bonfrate L., de Bari O., Wang D.Q., Portincasa P. (2012). A pleiotropic role for the orphan nuclear receptor small heterodimer partner in lipid homeostasis and metabolic pathways. J. Lipids.

[56] Katsuoka F., Motohashi H., Engel J.D., Yamamoto M. (2005). Nrf2 transcriptionally activates the mafG gene through an antioxidant response element. J. Biol. Chem..

[57] de Aguiar Vallim T.Q., Tarling E.J., Ahn H., Hagey L.R., Romanoski C.E., Lee R.G., Graham M.J., Motohashi H., Yamamoto M., Edwards P.A. (2015). MAFG is a transcriptional repressor of bile acid synthesis and metabolism. Cell Metab..

[58] Beltroy E.P., Richardson J.A., Horton J.D., Turley S.D., Dietschy J.M. (2005). Cholesterol accumulation and liver cell death in mice with Niemann-Pick type C disease. Hepatology.

[59] Gan L.T., Van Rooyen D.M., Koina M.E., McCuskey R.S., Teoh N.C., Farrell G.C. (2014). Hepatocyte free cholesterol lipotoxicity results from JNK1-mediated mitochondrial injury and is HMGB1 and TLR4-dependent. J. Hepatol..

[60] Ioannou G.N., Landis C.S., Jin G.Y., Haigh W.G., Farrell G.C., Kuver R., Lee S.P., Savard C. (2019). Cholesterol Crystals in Hepatocyte Lipid Droplets Are Strongly Associated with Human Nonalcoholic Steatohepatitis. Hepatol. Commun..

[61] Sozen E., Demirel-Yalciner T., Sari D., Ozer N.K. (2022). Cholesterol accumulation in hepatocytes mediates IRE1/p38 branch of endoplasmic reticulum stress to promote nonalcoholic steatohepatitis. Free Radic. Biol. Med..

[62] Song Y., Liu J., Zhao K., Gao L., Zhao J. (2021). Cholesterol-induced toxicity: An integrated view of the role of cholesterol in multiple diseases. Cell Metab..

[63] Reddy N.M., Potteti H.R., Mariani T.J., Biswal S., Reddy S.P. (2011). Conditional deletion of Nrf2 in airway epithelium exacerbates acute lung injury and impairs the resolution of inflammation. Am. J. Respir Cell Mol. Biol..

[64] Postic C., Shiota M., Niswender K.D., Jetton T.L., Chen Y., Moates J.M., Shelton K.D., Lindner J., Cherrington A.D., Magnuson M.A. (1999). Dual roles for glucokinase in glucose homeostasis as determined by liver and pancreatic beta cell-specific gene knock-outs using Cre recombinase. J. Biol. Chem..

[65] Han H., Tanigaki K., Yamamoto N., Kuroda K., Yoshimoto M., Nakahata T., Ikuta K., Honjo T. (2002). Inducible gene knockout of transcription factor recombination signal binding protein-J reveals its essential role in T versus B lineage decision. Int. Immunol..

[66] Wakabayashi N., Yagishita Y., Joshi T., Kensler T.W. (2023). Forced Hepatic Expression of NRF2 or NQO1 Impedes Hepatocyte Lipid Accumulation in a Lipodystrophy Mouse Model. Int. J. Mol. Sci..

[67] Chartoumpekis D.V., Palliyaguru D.L., Wakabayashi N., Fazzari M., Khoo N.K.H., Schopfer F.J., Sipula I., Yagishita Y., Michalopoulos G.K., O’Doherty R.M. (2018). Nrf2 deletion from adipocytes, but not hepatocytes, potentiates systemic metabolic dysfunction after long-term high-fat diet-induced obesity in mice. Am. J. Physiol. Endocrinol. Metab..

[68] Chartoumpekis D.V., Yagishita Y., Fazzari M., Palliyaguru D.L., Rao U.N., Zaravinos A., Khoo N.K., Schopfer F.J., Weiss K.R., Michalopoulos G.K. (2018). Nrf2 prevents Notch-induced insulin resistance and tumorigenesis in mice. JCI Insight.

