# Supplementary material for: Dual Deletion of Keap1 and Rbpjκ Genes in Liver Leads to Hepatomegaly and Hypercholesterolemia
Source: Int J Mol Sci. 2024 Apr 26;25(9):4712. doi: 10.3390/ijms25094712 (PMC11083431; doi:10.3390/ijms25094712)
Supplement: Supplementary file 1 [file ijms-25-04712-s001.zip › ijms-2984836-supplementary.pdf]

Supplementary Materials

Dual deletion of *Keap1* and *Rbpjκ* genes in liver leads to hepatomegaly and hypercholesterolemia

Nobunao Wakabayashi[1], Yoko Yagishita[1,2], Tanvi Joshi[1] and Thomas W. Kensler[1,*]

1 Translational Research Program, Fred Hutchinson Cancer Center, Seattle, WA 98109, USA.
nwakabay@fredhutch.org (N.W.); yyagishi@fredhutch.org or yy3328@cumc.columbia.edu (Y.Y.); tjoshi@fredhutch.org (T.J.); tkensler@fredhutch.org (T.W.K.)
2 Division of Endocrinology, Columbia University, New York, NY 10032, USA
* Correspondence: tkensler@fredhutch.org

[number] corresponds to references number in the article.

Table S1. Primers for mouse genotyping

Genotyping for Rbpjκ$^{F/F}$ background mouse	
**Primers**	**Sequence (5' - 3')**
Rbpjk-Int6.2	CCTTTCTTTGTGCGTGCCTCC
Rbpjk-Ex7.2	TGACAGTCTGCCCGTAATGG
Rbpjk-Int8.2	CCTAGAACAGGCTGCCTGATCACCCTTCC

Genotyping for Keap1$^{F/F}$ background mouse [66]	
5-cko 4int1	GCACATCCTTCATCTCTCCGCACTGGGGAG
3-Kp1-4Ex	CCTCCGTGTCAACATTGGCGCGACTAG
R260-EGFP	GACTTGAAGAAGTCGTGCTGCTTCATGTG

Genotyping for Nrf2$^{F/F}$ background mouse [67]	
N3	TGAGAGCTTCCCAGACTCACTT
mNrf2Ex-V-32	CTGGGCTGGGAACAGCGGTAGTATCAGCCAGC

Genotyping for Cre background mouse [22]	
Cre1	ACGTTCACCGGCATCAACGT
Cre2	CTGCATTACCGGTCGATGCA

Table S2. PCR programs for mouse genotyping

Keap1$^{F/F}$ background mouse [66]			
**Step #**	**Temp °C**	**Time**	**Note**
1	95	1 min	-
2	95	30 sec	-
3	68.5	30 sec	-
4	72	30 sec	repeat steps 2-4 for 35 cycles
5	4	-	hold
Product; Flox : ~350 bp, Disrupted : ~550 bp, Wt : ~250 bp			

Rbpjκ$^{F/F}$ background mouse			
**Step #**	**Temp °C**	**Time**	**Note**
1	95	1 min	-
2	95	30 sec	-
3	68.5	30 sec	-
4	72	30 sec	repeat steps 2-4 for 35 cycles
5	72	1 min	-
6	4	-	hold
Product; Flox : ~515 bp, Disrupted : ~300 bp, Wt : ~415 bp			

Table S3. Primers for deletion check genotyping of *Nrf2$^{F/F}$* mouse and its PCR program

Genotyping for Nrf2$^{F/F}$ background mouse [63]	
N1	TCTTAGGCACCATTTGGGAGAG
N2	TACAGCAGGCATACCATTGTGG
N3	TGAGAGCTTCCCAGACTCACTT

Nrf2$^{F/F}$ background mouse			
**Step #**	**Temp °C**	**Time**	**Note**
1	95	1 min	-
2	95	30 sec	-
3	68.7	30 sec	-
4	72	30 sec	repeat steps 2-4 for 35 cycles
5	4	-	hold
Product; Flox : ~750 bp, Disrupted : ~405 bp			

Table S4. Primers for the real-time PCR analyses.

**Gene**	**Forward**	**Reverse**
Srebp1c	GGAGCCATGGATTGCACATT	GGCCCGGGAAGTCACTGT
Acc1	ATGGGCGGAATGGTCTCTTTC	TGGGGACCTTGTCTTCATCAT
Fasn	GGAGGTGGTGATAGCCGGTAT	TGGGTAATCCATAGAGCCCAG
Scd1	TTCTTGCGATACACTCTGGTGC	CGGGATTGAATGTTCTTGTCGT
Srebp2	CGAGCAACGGGACCATTCT	CCCCATGACTAAGTCCTTCAACT
Hmgcs1	GCAGTCTTCAATGCCGTGAA	GCAATGTCTCCTGCAACTACCA
Hmgcr	TTGGTCCTTGTTCACGCTCAT	TTCGTCCAGACCCAAGGAAAC
Mvd	ATGGCCTCAGAAAAGCCTCAG	TGGTCGTTTTTAGCTGGTCCT
Ldlr	CCAATCGACTCACGGGTTCA	TCACACCAGTTCACCCCTCT
Nr0b2	CAGGTCGTCCGACTATTCTGT	AGGCTACTGTCTTGGCTAGGA
Cyp7A1	AGCAACTAAACAACCTGCCAGTACTA	GTCCGGATATTCAAGGATGCA
Abcb11	CAGGGAGGCCAAAGGTGAGC	ATGGTGGCAGGGAATGAAAAGTAG
18S rRNA	CTCAACACGGGAAACCTCAC	CGCTCCACCAACTAAGAACG

[67,68] and https://pga.mgh.harvard.edu/primerbank/

Table S5. Antibodies used in this research.

Target Protein	Provider	Dilution
Nrf2	Invitrogen PA5-27882	2,000
Keap1	Original	3,000
Rbpjκ	Santa Cruz Bio sc-28713	500
NR0B2	Invitrogen PA5-76632	500
LaminB1	Proteintech 12987-1-AP	5,000
Nqo1	Abcam ab2346	500
GstA1-5	Invitrogen PA5-79335	2,000
Mvd	Proteintech 15331-1-AP	1,000
SREBPF2	Proteintech 28212-1-AP	2,000
HMGCS1	Cell Signaling Tech Q01581	1,000
CYP7A1	Invitrogen PA5-100892	500
Rabbit anti-Goat IgG (H+L)-HRP	Invitrogen 31402	10,000
Goat Anti-Rabbit IgG (H + L)-HRP	BIO RAD 1706515	3,000

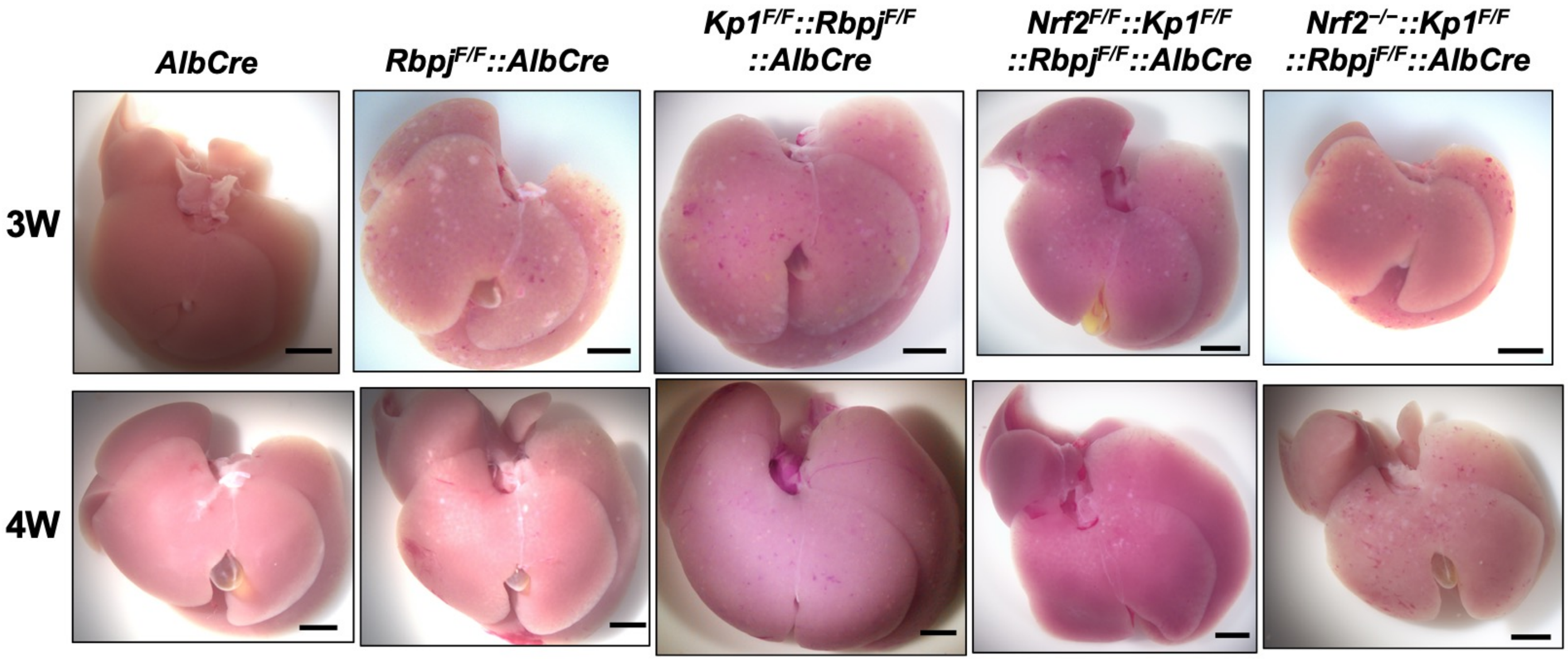

Figure S1. Changes in liver morphology among the genotypes. Representative livers from 3W and 4W old males. The scale bar = 5 mm.

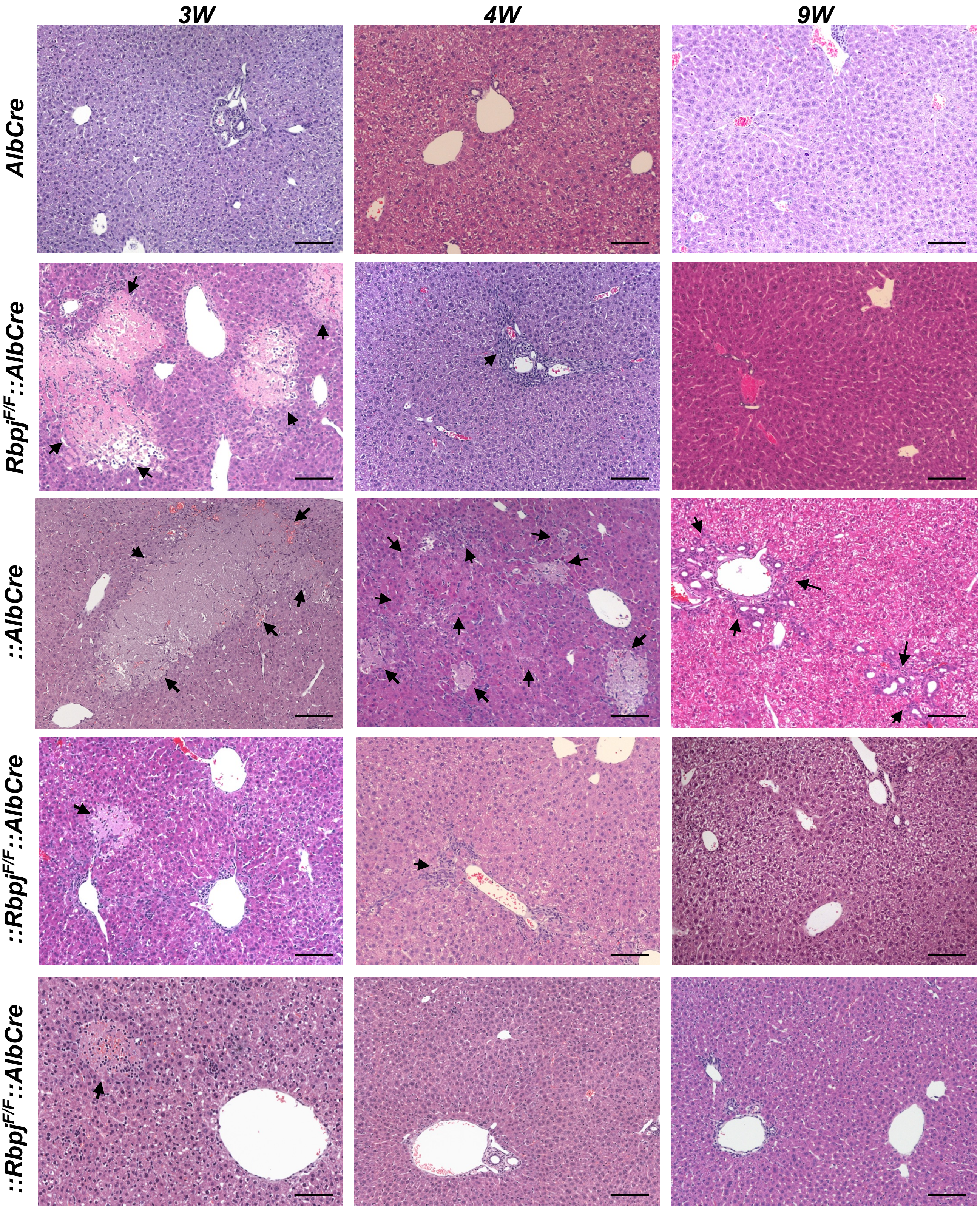

Figure S2. Changes in liver morphology among the genotypes. H&E stained sections from the central lobe of each genotype mouse at 3W, 4W and 9W of age. Arrows indicate the degeneration and damage produced by cholestasis. The scale bar = 100 µm.

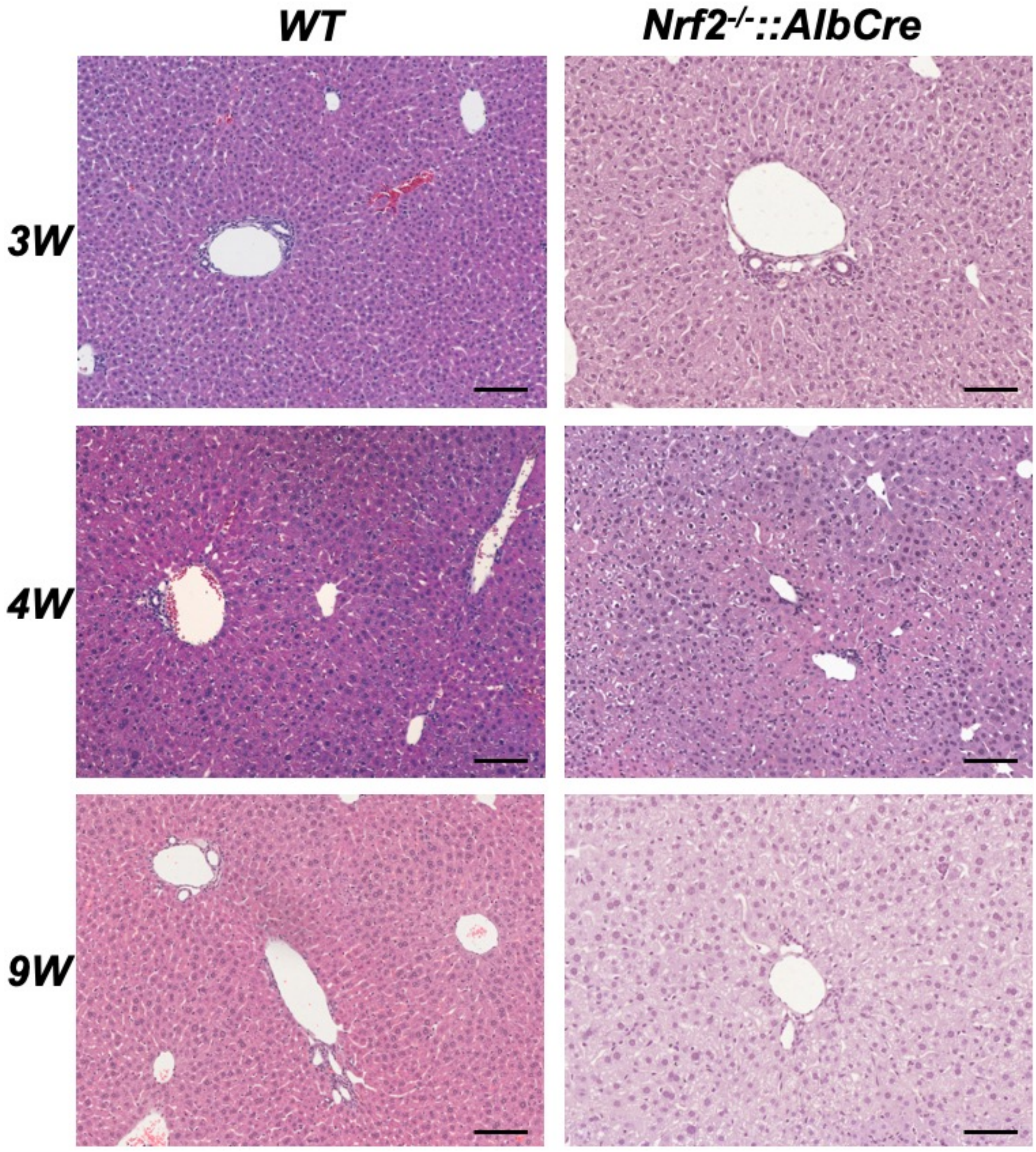

Figure S3. H&E stained sections from the central lobe of control genotype mice at 3W, 4W and 9W of age. The scale bar = 100 μm.

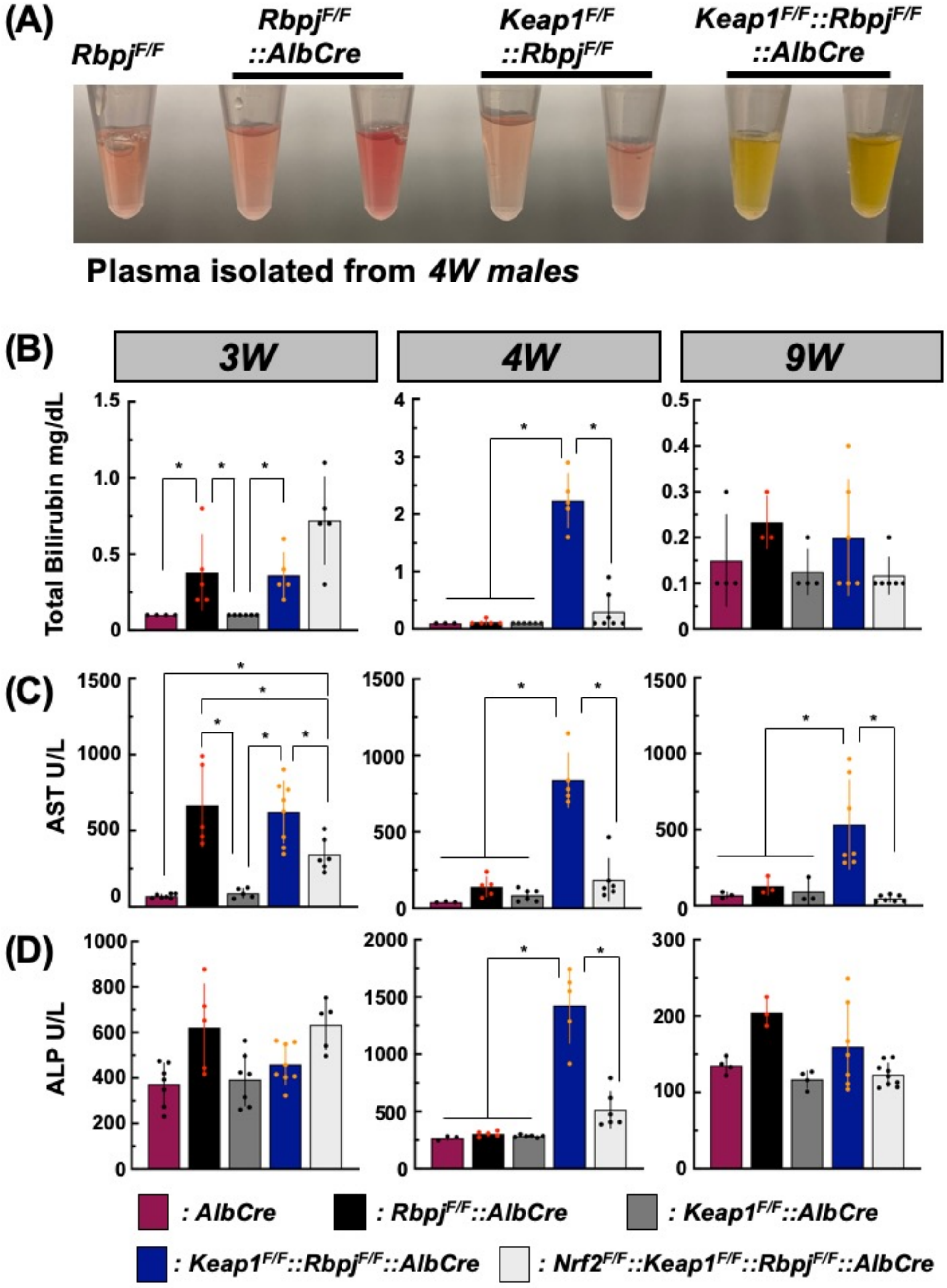

Figure S4. Features of plasma color (4W) and plasma biochemical analyses (3W, 4W and 9W of age) in the relevant mouse genotypes. N=3-9 * $p < 0.05$, by Tukey's.

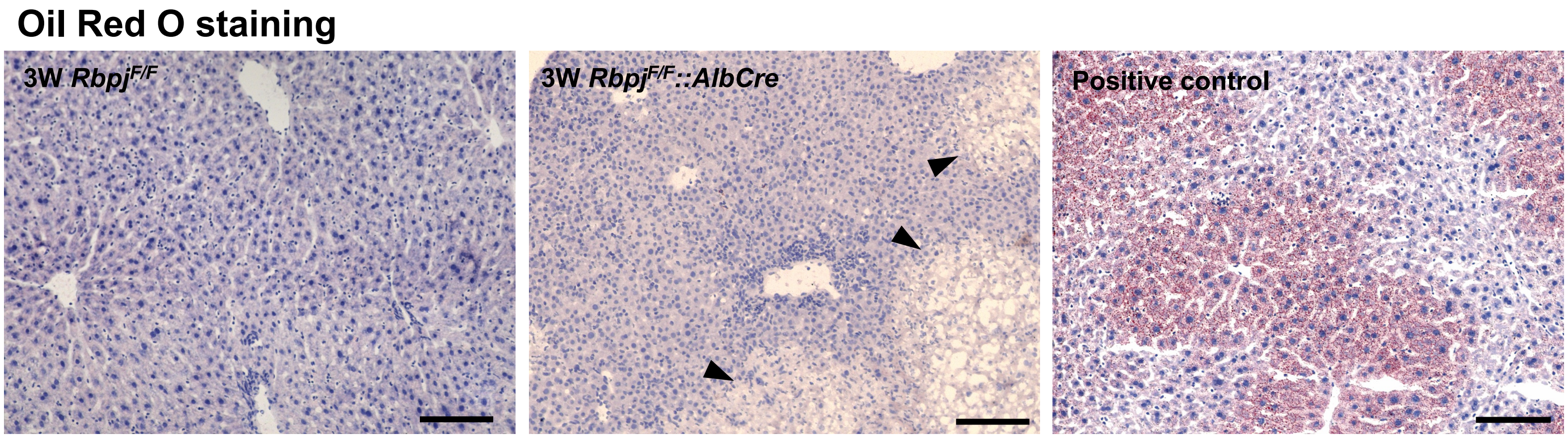

Figure S5. The degenerated foci observed in *Rbpj*$^{F/F}$*::AlbCre* liver were confirmed by oil red O staining. Arrow heads indicate degenerated regions in the liver. The scale bar = 100 µm.

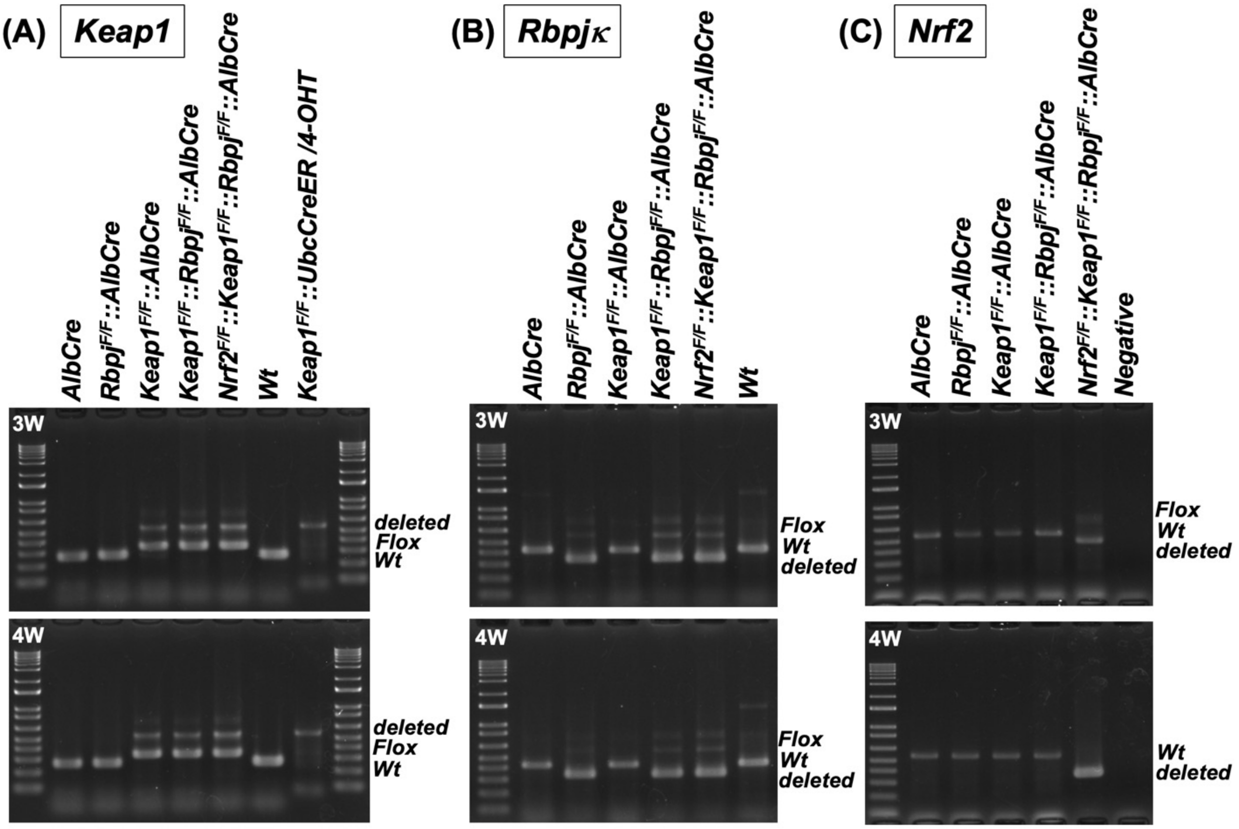

Figure S6. Representative profiles of gene allelic conversions by *AlbCre* expression. *Keap1*, *Rbpjκ* and *Nrf2* allelic change are depicted in (A), (B) and (C), respectively. The top and bottom panels show PCR results from 3W and 4W mouse liver genomic DNA as template, respectively. The PCR conditions for detection of *Keap1* and *Nrf2* conversion have been described previously[63], [66]. For *Rbpjκ*, PCR conditions and its primers are shown in Table S1, S2 and Figure S4.

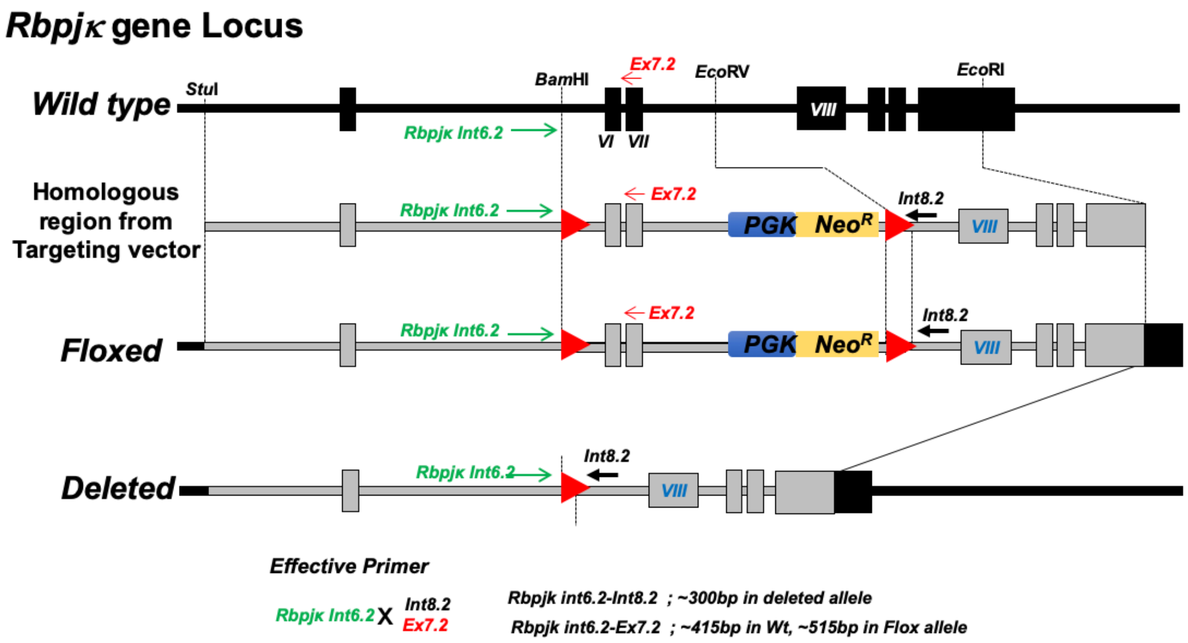

Figure S7. Mouse *Rbpjκ* and its flox mutant gene structure and primer positions for genotyping. Positions for each primer for Int6.2, Int8.2 and Ex 7.2 are indicated by green, black and red arrows, respectively. Red triangles indicate LoxP elements. The structures provided arise from the original paper[65].

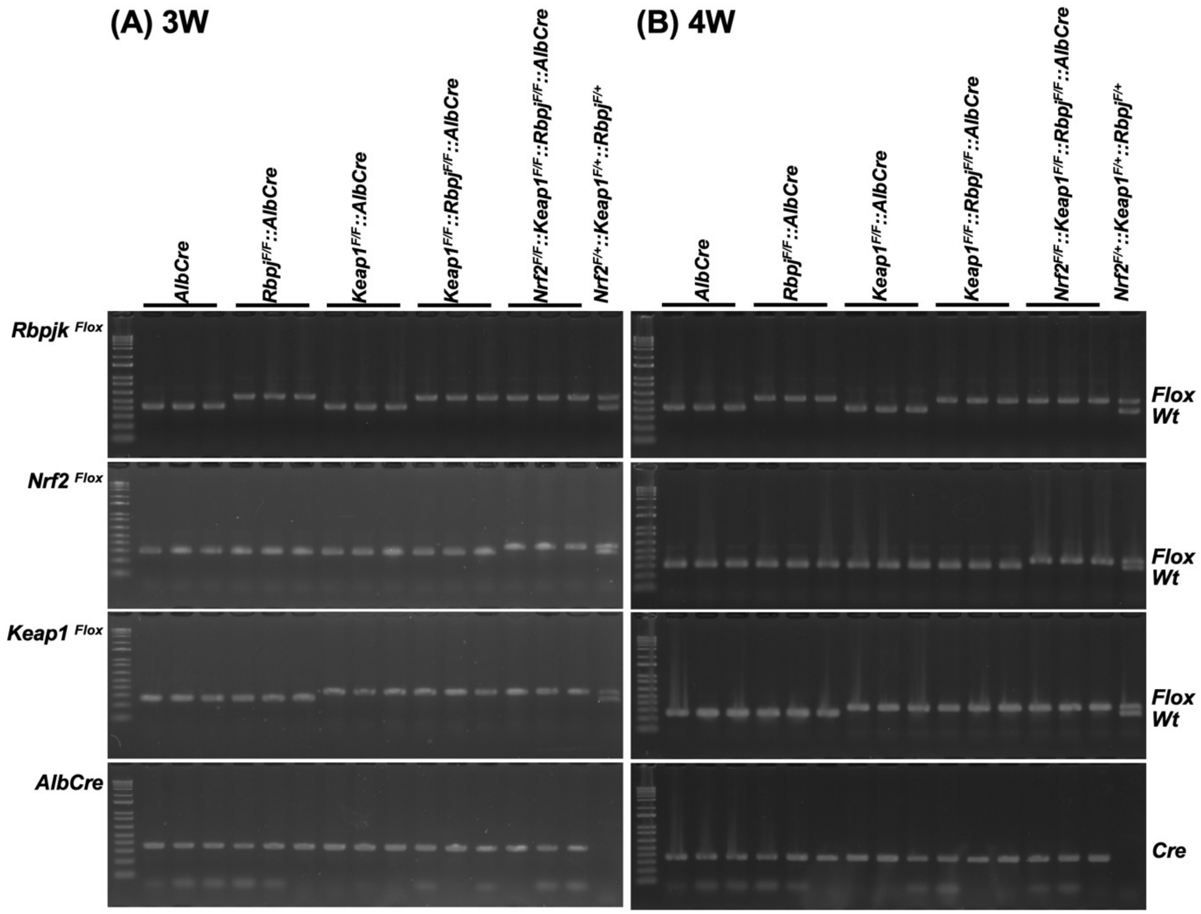

Figure S8. Representative results of confirmatory genotyping. DNA isolated from tail snips of each heterozygote of *Nrf2*$^{F/+}$*::Keap1*$^{F/+}$*::Rbpjk*$^{F/+}$ and *Rbpjk*^{Flox} (top), *Nrf2*^{Flox} (second), *Keap1*^{Flox} (third), and *Albumin Cre* (bottom) was used for genotyping. Analyses from 3W and 4W old mice are presented in panels (A) and (B), respectively.

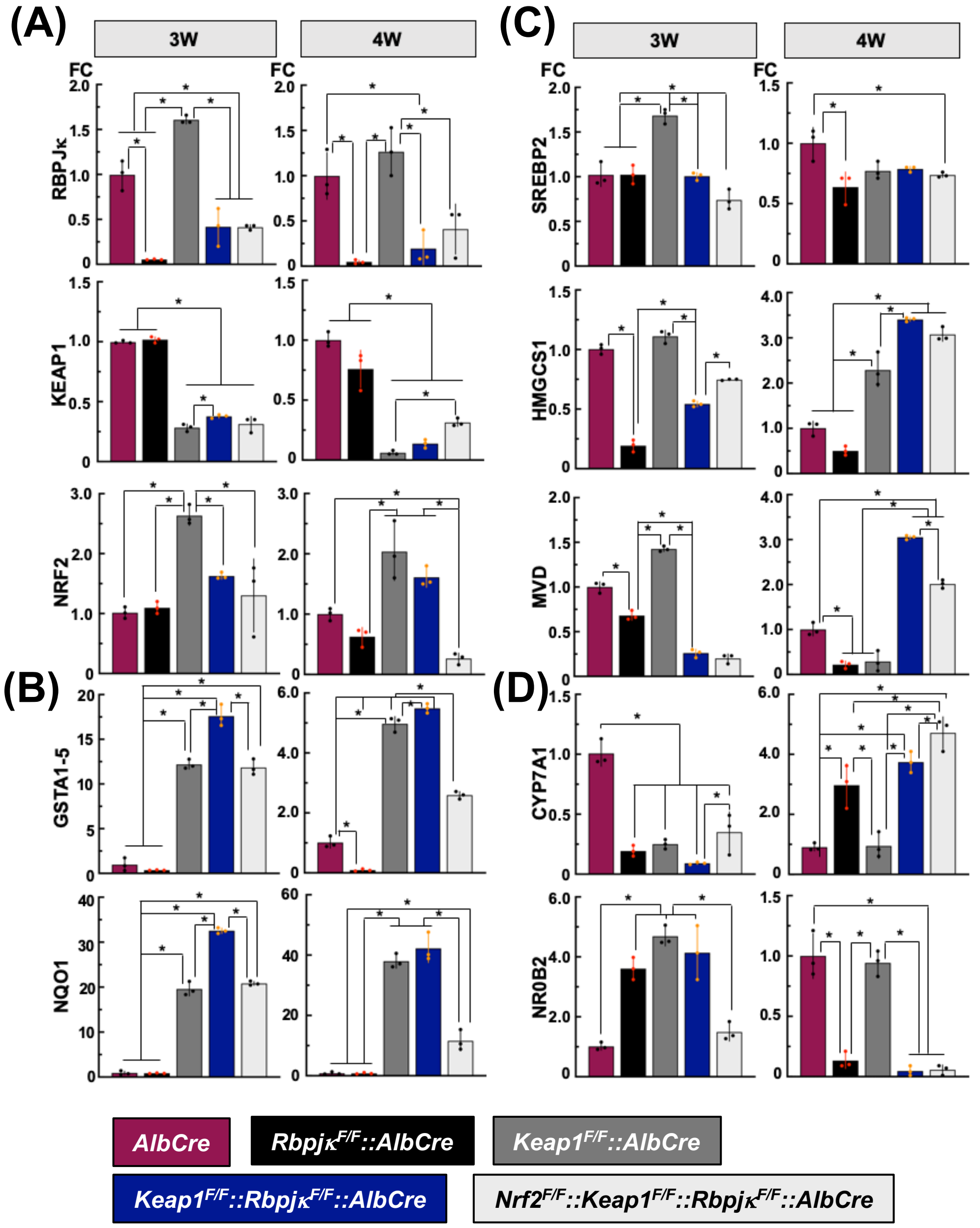

Figure S9. Immunoblotting analyses of putative ARE-regulated (NRF2) gene products in metabolic pathways affecting cholesterol-bile acid flux in 3W and 4W old mice. Liver extracts from 3 individual mice for each genotype were examined; quantification was performed by normalizing with LMNB1 expression. Deletion target gene products (A), representative NRF2 target genes (B), cholesterol synthesis related gene products (C) and bile acid pathway related gene products (D) are presented. The Y axis indicates the level of fold change (FC) where gene expression in *AlbCre* mice was set as 1. N=3. * $p < 0.05$ by Tukey's.
